# Supplementary figures and images for: Research on using Aquilaria sinensis callus to evaluate the agarwood-inducing potential of fungi
Source: PLoS One. 2024 Dec 26;19(12):e0316178. doi: 10.1371/journal.pone.0316178 (PMC11671001; doi:10.1371/journal.pone.0316178)

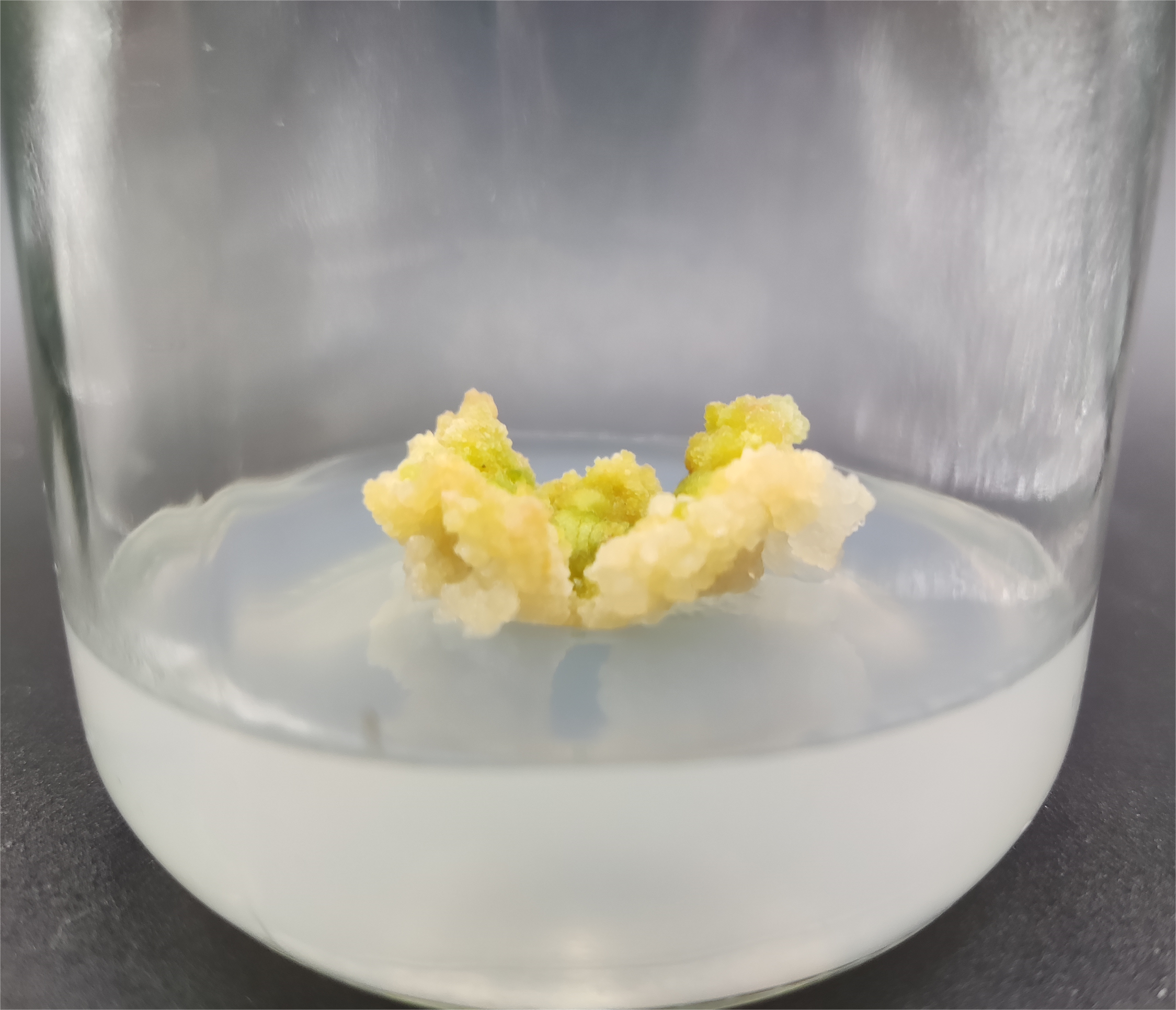

Supplement: S1 Fig — (TIF) [file pone.0316178.s001.tif]
