# Supplementary material for: Research on using Aquilaria sinensis callus to evaluate the agarwood-inducing potential of fungi
Source: PLoS One. 2024 Dec 26;19(12):e0316178. doi: 10.1371/journal.pone.0316178 (PMC11671001; doi:10.1371/journal.pone.0316178)
Supplement: S1 Table — (PDF) [file pone.0316178.s002.pdf]

S2 Table. GS-MS results of W-1 treatment.

No.	Retention Time (min)	Compound	Relative amount / %
**Sesquiterpenes**			**9.43**
82	24.33	alpha-Curcumene	0.06
99	27.79	3,3,6,6,9,9-Hexamethyltetracyclo [6.1.0.02,4.05,7] nonane	0.11
100	27.86	beta-Patchoulene	0.04
101	27.94	4a,5-dimethyl-3-prop-1-en-2-yl-2,3,4,5,6,7-hexahydro-1H-naphthalene	0.22
102	28.04	(-)-Aristolene	0.14
104	28.23	alpha-Patchoulene	0.13
113	29.53	Diepicedrene-1-oxide	0.04
119	30.1	1H-Cycloprop[e]azulene, decahydro-1,1,4,7-tetramethyl-	0.06
123	30.56	Solavetivone	0.26
124	30.61	Dehydrofukinone	0.14
129	31.25	beta-Selinene	0.47
134	31.71	Alloaromadendrene	0.74
135	31.8	2-(4a,8-Dimethyl-1,2,3,4,4a,5,6,7-octahydro-naphthalen-2-yl)-prop-2-en-1-ol	0.36
136	31.89	Bicyclo[5.3.0]decane, 2-methylene-5-(1-methylvinyl)-8-methyl-	0.18
137	31.98	Z-beta-Guaiene	0.54
145	33.01	Aromandendrene	0.21
146	33.05	(+)-Eremophilene	0.27
148	33.27	Cycloheptane, 4-methylene-1-methyl-2-(2-methyl-1-propen-1-yl)-1-vinyl-	1.54
149	33.32	(-)-cis-beta-Elemene	1.48
152	33.56	beta-Ionone	0.23
158	34.02	5-Octen-2-one, 6-methyl-8-(2,6,6-trimethyl-1-cyclohexen-1-yl)-	0.11
161	34.42	Longifolenaldehyde	1.25
167	34.85	Caryophyllene oxide	0.14
208	39.77	Squalene	0.34
218	41.65	Cholestan-3-ol, 6-methyl-, (3.beta.,5.alpha.,6.alpha.)-	0.37
**Aromatic compounds**			**21.61**
15	6.62	2,4,5-Trichlorophenyl cinnamate	0.07
24	9.74	Benzoic acid, p-tert-butyl-	0.01
25	9.85	Benzaldehyde	0.15
26	9.93	Silane, diethyl(2-phenylethoxy)propoxy-	0.01
34	11.54	2,4,6-Cycloheptatrien-1-one, 2-hydroxy-	0.01
40	12.25	Phenol, 2,6-dimethyl-	0.01
43	12.94	Acetophenone	0.01
54	15.78	Benzamide, 3-amino-	0.01

(Continued)

S2 Table. (Continued)

55	15.94	Phthalic acid, 2-ethoxyethyl ethyl ester	0.05
56	16.54	2H-Indol-2-one, 1,3-dihydro-5-hydroxy-	0.02
60	18.12	2-Butanone, 4-phenyl-	2.28
61	18.24	1-(2,3-Dimethylphenyl)ethanone	0.01
63	18.41	Benzaldehyde, 3-methoxy-	0.04
66	19.77	2-Chloro-4-fluoroaniline	0.01
70	20.65	Hydrocinnamic acid	0.15
71	20.9	Benzenepropanoic acid, ethyl ester	0.03
75	21.2	(3-Methoxyphenyl)acetonitrile	0.01
76	21.25	2H-Indol-2-one, 3-ethyl-1,3-dihydro-1-methyl-	0.01
81	24.23	Benzenamine, N,N-diethyl-4-(2-nitroethenyl)-	0.05
83	24.49	2-Benzyl-3-methoxycyclopropanecarboxylic acid	0.05
84	24.85	2-Butanone, 4-(4-methoxyphenyl)-	4.28
89	26.11	2-Butanone, 4-(4-hydroxyphenyl)-	0.03
91	26.45	7-Benzofuranol, 2,3-dihydro-2,2-dimethyl-	0.11
93	26.71	3-(4-Methoxyphenyl)propionic acid	0.18
105	28.54	Butan-2-one, 4-(3-hydroxy-2-methoxyphenyl)-	0.92
106	28.63	2-Butanone, 4-(4-hydroxy-3-methoxyphenyl)-	0.11
107	28.75	3,4-Dimethoxyphenylacetone	0.04
108	28.86	2,4-Pentanedione, 3-(phenylmethyl)-	0.11
109	28.96	1-(4-tert-Butylphenyl)propan-2-one	0.18
112	29.41	Acetic acid, trifluoro-, 2-methoxyphenyl ester	0.2
114	29.62	4-Methoxyphenylacetic acid, hydrazide	0.14
116	29.85	Phenol, 2-ethyl-4,5-dimethyl-	0.06
118	30.03	Pyrrolo[2,3-b]indole, 1,2,3,3a,8,8a-hexahydro-5-methoxy-3a,8-dimethyl-,	0.09
132	31.5	4-Fluoro-4'-hydroxybenzophenone	0.16
140	32.4	Dibutyl phthalate	0.26
143	32.76	Thiourea, 1-(2,4,6-trimethylphenyl)-3-(2-propynyl)-	1.31
147	33.14	1H-Imidazole, 2-methyl-4-nitro-1-phenyl-	0.25
153	33.64	2H-Benz[e]indene-3,7-diol, 3,3a,4,5-tetrahydro-3a-methyl-, (3S-cis)-	0.73
155	33.75	Spiro[2,3-dihydroindol-3,5'-2'-thiazoline], 2'-methylthio-2-oxo-	0.33
157	33.95	Naphthalene, 5-ethyl-1,2,3,4-tetrahydro-	0.15
164	34.64	1-Penten-3-one, 1,5-diphenyl-	0.16
180	35.93	Benzene, 1,3-bis(1-buten-3-yl-)-	0.09
181	36.14	Butanedioic acid, butyl phenylmethyl ester	0.07
182	36.27	1-Benzyl-5-methylsulfanyl-3-nitro-1H-[1,2,4]triazole	1.3
184	36.51	Acetamide, N-methyl-N-(4-methylphenyl)-	0.05
185	36.63	1,5-Diphenylhex-3-ene	0.02
187	36.8	Acetamide, N-(4-benzyloxyphenyl)-2-cyano-	0.08

(Continued)

S2 Table. (Continued)

191	37.38	4-Benzyloxybenzophenone	0.03
192	37.5	1-Penten-3-one, 4,4-dimethyl-1-phenyl-	0.01
194	37.73	Phthalic acid, di(2-propylpentyl) ester	0.07
195	37.96	Carbonic acid, propargyl 4-benzyloxyphenyl ester	0.17
196	38.11	2-(Salicylideneamino)naphthalene	0.02
197	38.19	4-Methoxybenzyl phenyl carbonate	0.03
198	38.38	9H-Benzo[4,5]imidazo[2,1-c][1,2,4]triazole, 3-benzylsulfanyl-	0.47
199	38.42	Benzeneacetic acid, (4-methoxyphenyl)methyl ester	0.3
200	38.52	trans-1-Cinnamoylimidazole	0.04
201	38.63	Benzene, (1-chloro-3-iodopropyl)-	0.02
203	38.87	Silane, dimethyl(4-methoxybenzyloxy)pentyloxy-	0.12
205	39.19	Pentanal, 2-[bis(phenylmethyl)amino]-4-methyl-	0.88
207	39.57	2-Benzazol-1,3-dione, N-(2-hydroxy-phenoxymethyl)-	0.1
209	40.08	4-(2-Mercapto-4-phenyl-imidazol-1-yl)-benzoic acid methyl ester	0.16
210	40.19	N-(2-Hydroxy-propionyl)-4-methyl-benzenesulfonamide, O-tert.-butyldimethylsilyl-	0.09
211	40.35	5-Fluoro-1,3-bis[phenylmethyl]-2,4(1H,3H)-pyrimidinedione	1.67
213	40.62	Quinazolin-4(3H)-one, 2-(4-methoxybenzylthio)-3-methyl-	0.66
214	40.92	Imidazolidin-4-one, 5-benzyl-2-thioxo-3-p-tolyl-	0.61
215	41.15	4-Methoxybenzylamine, N,N-dihexyl-	0.7
219	41.79	(4-Methoxy-benzyl)-phenethyl-amine	0.08
220	42.02	Benzene, 1-trifluoromethyl-4-(3-methoxybenzyloxy)-3-nitro-	0.11
221	42.12	(.+/-.)-p-Methoxyamphetamine, N-pentafluoropropionyl-	0.04
222	42.47	1H-[1,2,3]Triazole-4-carboxamide, 5-amino-N-(4-fluorophenyl)-1-(4-methoxybenzyl)-	0.38
223	42.61	Benzeneacetic acid, .alpha.-methoxy-, methyl ester, (.+/-.)-	0.13
224	43.34	Salicylhydrazide,N2-[1-methyl-3-(2,6,6-trimethylcyclohex-2-enyl)prop-2-enylideno]-	0.19
225	43.54	Anisole, p-octyl-	0.06
227	44.58	Benzimidazole, 5-(4-methoxybenzylamino)-1-phenyl-	0.05
228	44.73	Glutaric acid, 2-methylhex-3-yl 2-methoxybenzyl ester	0.02
**Chromones**			**3.06**
67	20.2	Phthalic anhydride	0.01
86	25.45	2-Methylchromone	0.02
88	26.02	1,3-Cyclohexanedione, 2-(phenylmethyl)-	0.11
188	37.04	1-(5-Hydroxy-6-hydroxymethyl-tetrahydropyran-2-yl)-5-methyl-1H-pyrimidine-2,4-dione	0.23
202	38.8	Coumarin, 6-benzyloxy-3,4-dihydro-4,4-dimethyl-	1.32
204	39.07	3-[(2-Methoxy-5-nitro-benzylidene)-amino]-2-methyl-3H-quinazolin-4-one	0.06
217	41.51	2,8-Dihydroxynaphthoquinone	1.11

(Continued)

S2 Table. (Continued)

226	43.88	2-Oxazolidinone, 4-phenyl-5-p-tolyl-, trans-	0.07
230	46.48	Stigmast-4-en-3-one	0.08
231	48.72	3-(2-Ethyl-piperidin-1-ylmethyl)-8a-methyl-5-methylene-decahydro-naphtho[2,3-b]furan-2-one	0.05
		**Alkanes**	**58.13**
1	3.13	Ethanol, 2-(vinyloxy)-	0.14
2	3.45	Ethyl Acetate	2.93
4	4.16	Ethane, 1,1-diethoxy-	0.13
5	4.42	1-Butaneboronic acid	4.98
6	4.55	Methane, isothiocyanato-	3.56
7	4.8	Propanamide, N,N-dimethyl-	1.33
8	4.98	1,3-Dioxolane, 2,4,5-trimethyl-	1.38
9	5.15	Silane, diethoxydimethyl-	0.13
10	5.52	N-(2-Methoxyethyl)-N-ethylnitrosamine	1.16
11	5.96	2,3-Butanediol	8.4
12	6.08	2,3-Butanediol, [R-(R*,R*)]-	4.53
13	6.38	Butane, 2-ethoxy-	2.17
14	6.51	Diethyl 2,2'-(2,2'-oxybis(ethane-2,1-diyl)bis(oxy))diacetate	0.15
16	6.74	Silane, butoxytrimethyl-	0.1
17	7.14	2-Propanol, 1,3-dimethoxy-	0.01
18	7.39	Acetic acid, methoxy-, methyl ester	0.68
19	7.61	1,3-Butanediol	0.13
20	7.85	Ethyl 2-(2-(2-ethoxyethoxy)ethoxy)acetate	0.01
21	7.97	2,3-Butanediol, diacetate	0.07
22	8.56	2-Ethoxy-3-chlorobutane	0.02
23	9.15	Acetic acid, methoxy-, ethyl ester	0.56
27	10.11	Propane, 2-ethoxy-	0.03
28	10.62	Diethyl carbonate	0.01
29	10.89	1,3-Dioxolane, 2-methoxymethyl-2,4,5-trimethyl-	0.04
30	10.99	Butanoic acid, 3-hydroxy-, ethyl ester	0.01
31	11.14	(2S,4S)-(+)-Pentanediol	0.02
32	11.39	4-Methoxymethoxy-3-nitro-pentan-2-ol	0.01
33	11.47	3,6,9-Trioxa-2-silaundecane, 2,2-dimethyl-	0.01
35	11.7	Formic acid, hexyl ester	0.01
36	11.79	1,3-Dioxolane, 2-ethyl-	0.02
37	11.99	2-Hexen-1-ol, 5-[[(1,1-dimethylethyl)dimethylsilyl]oxy]-, (E)-(.+-.)-	0.04
38	12.07	(Methoxymethyl)trimethylsilane	0.01
39	12.14	1,1,3-Trimethoxypropane	0.01
41	12.31	Ethyl(dimethyl)ethoxysilane	0.04
42	12.69	2-Propanone, 1-methoxy-	0.02

(Continued)

S2 Table. (Continued)

44	13	1H-1,2,4-Triazole, 3-thiol-5-methyl-	0.01
45	13.07	Butanoic acid, 3-hydroxy-, methyl ester	0.01
46	14.39	3-Hexene, 1-(1-ethoxyethoxy)-, (Z)-	0.01
47	14.66	1-Propanol, 2-methyl-2-nitro-	0.03
48	14.86	1,3-Dioxolane, 2-(dichloromethyl)-	0.01
49	14.98	1,3-Dioxolane, 2-(chloromethyl)-	0.01
50	15.29	1,3-Dioxolane, 2-(1-methylethyl)-	0.04
51	15.39	2-[2-(2-Ethoxyethoxy)ethoxy]ethoxy-trimethylsilane	0.05
52	15.56	Boronic acid, ethyl-, dimethyl ester	0.01
53	15.71	3-Nitropropanoic acid	0.01
57	16.7	Dimethyl diglycolcarbonate	0.01
58	16.96	3,3-Diethoxy-1-propanol	0.03
59	17.09	Silane, dimethyl(dimethyl(undec-2-enyloxy)silyloxy)ethoxy-	0.08
62	18.33	Benzene, 1,3-bis(1,1-dimethylethyl)-	0.02
64	19.51	1,3,5-Triazine-2,4-diamine, 6-chloro-	0.01
65	19.56	Butyric acid, tert-butyldimethylsilyl ester	0.01
68	20.36	1-Pentamethyldisilyloxybutane	0.01
69	20.48	2-Methyl-1-pentamethyldisilyloxypropane	0.01
72	20.98	2-Pentamethyldisilyloxypropane	0.01
73	21.03	6-Ethyl-3-pentamethyldisilyloxydecane	0.01
74	21.12	2-Pentamethyldisilanyloxybutane	0.01
77	21.93	1H-1,3a-Ethanopentalen-5(4H)-one, 2,3-dihydro-	0.01
78	22.34	Silane, (2-ethoxyethoxy)trimethyl-	0.01
79	23.16	3-(2-Methylphenyl)propionic acid	0.11
80	24.13	Silane, dimethyl(dimethyl(but-2-enyloxy)silyloxy)(but-2-enyloxy)-	0.01
85	24.99	Phenol, 2,4-bis(1,1-dimethylethyl)-	0.15
87	25.91	4-Methoxycarbonylbutyl-1-methyl-2-methoxycarbonylethyl ether	0.01
90	26.34	Triisopropylsilyloxycyclobutane	0.03
92	26.61	Cyclohexane, 1,5-diethenyl-2,3-dimethyl-, (1.alpha.,2.alpha.,3.alpha.,5.beta.)-	0.03
94	27.1	2,3-Dimethylphenoxyacetic acid	0.01
95	27.18	7-Propylidene-bicyclo[4.1.0]heptane	0.02
96	27.51	Hepta-2,6-dienoic acid, 2-methoxy-5-methylene-, methyl ester	0.01
97	27.61	3-Methoxymethoxy-3,7,16,20-tetramethyl-heneicosa-1,7,11,15,19-pentaene	0.06
98	27.73	3-(2-Methoxymethoxyethylidene)-2,2-dimethylbicyclo[2.2.1]heptane	0.01
103	28.18	Naphthalene,decahydro-4a-methyl-1-methylene-7-(1-methylethylidene)-, (4aR-trans)-	0.16
110	29.05	Bicyclo[6.3.0]undeca-1(8),9-diene, 11,11-dimethyl-	0.09

(Continued)

S2 Table. (Continued)

111	29.23	o-Mentha-1(7),8-dien-3-ol	0.08
115	29.71	Tetradecanoic acid	0.06
117	29.91	1,1,6-trimethyl-3-methylene-2-(3,6,9,13-tetramethyl-6-ethenye-10,14-dimethylene-pentadec-4-enyl)cyclohexane	0.16
120	30.29	Neoclovene oxide	0.13
121	30.35	Fumaric acid, di(2-ethylcyclohexyl) ester	0.14
122	30.5	1H-Indene, 1-ethylideneoctahydro-7a-methyl-, (1Z,3a.alpha.,7a.beta.)-	0.15
125	30.85	4,6,6-Trimethyl-2-(3-methylbuta-1,3-dienyl)-3-oxatricyclo[5.1.0.0(2,4)]octane	0.14
126	30.95	3,3-Diisopropylazetidin-2,4-dione	0.21
127	31.04	5-Aminomethylene-6-hydroxy-4-methyl-2-oxo-2,5-dihydro-3-pyridinecarbonitrile	1.06
128	31.12	(-)-Isolongifolol, methyl ether	0.21
130	31.36	Adipic acid, diphenyl ester	0.09
131	31.43	1-(2-Furyl)-3-(2-thienyl)-2-propen-1-one	0.18
133	31.64	Cyclohexene, 1-(2-methyl-2-cyclopenten-1-yl)-	0.77
138	32.14	2(1H)-Naphthalenone, 4a,5,6,7,8,8a-hexahydro-6-[1-(hydroxymethyl)ethenyl]-4,8a-dimethyl-, [4ar-(4a.alpha.,6.alpha.,8a.beta.)]-	0.96
139	32.34	n-Hexadecanoic acid	1.17
141	32.56	Pyrazole, 1,3,5-trimethyl-4-(3,5-dimethyl-4-pyrazolylazo)-	1.29
142	32.64	Hexadecanoic acid, ethyl ester	0.24
144	32.83	Bicyclo[4.1.0]heptan-2-one, 3,4,4-trimethyl-3-(3-methyl-1,3-butadienyl)-, [1.alpha.,3.alpha.(E),6.alpha.]-(.+-.)-	0.34
150	33.44	6-(1-Hydroxymethylvinyl)-4,8a-dimethyl-3,5,6,7,8,8a-hexahydro-1H-naphthalen-2-one	1.08
151	33.51	Cyclopropane, 1-(2-bromo-3-methyl-1-pentenylidene)-2,2,3,3-tetramethyl-	0.21
154	33.7	2-Methyl-5-nitro-2H-indazole	0.26
156	33.83	Methyl 5,7-hexadecadiynoate	0.23
159	34.17	9-Octadecenoic acid, (E)-	0.81
160	34.36	Octadecanoic acid	0.45
162	34.51	Ethanone, 1-[2-(5-hydroxy-1,1-dimethylhexyl)-3-methyl-2-cyclopropen-1-yl]-	0.1
163	34.58	Hexadecanamide	0.12
165	34.72	Cyclohexanecarboxylic acid, 2-hydroxy-, ethyl ester	0.04
166	34.77	4,4-Dimethyl-3-(3-methylbut-3-enylidene)-2-methylenebicyclo[4.1.0]heptane	0.11
168	34.91	Dodecanoic acid, 3,7,11-trimethyl-, methyl ester	0.14
169	35.03	Murolan-3,9(11)-diene-10-peroxy	0.14

(Continued)

S2 Table. (Continued)

170	35.15	Succinic acid, 10-chlorodecyl octyl ester	0.2
171	35.19	Succinic acid, dodec-9-yn-1-yl propyl ester	0.03
172	35.24	Succinic acid, butyl 8-chloroctyl ester	0.14
173	35.32	Ethyl hydrogen succinate	0.15
174	35.42	4-Bromo-3-oxobutyric acid, methyl ester	0.04
175	35.5	Tetracyclo[5.2.1.0(2,6).0(3,5)]decane, 4,4-dimethyl-	0.09
176	35.6	Ethyl 4,8,12-trimethyl-tridecanoate	0.06
177	35.7	Succinic acid, butyl tetradecyl ester	0.08
178	35.76	1-(1-Butoxypropan-2-yloxy)propan-2-yl acetate	0.04
179	35.82	8-Chlorooctyl (E)-2-methylbut-2-enoate	0.13
183	36.41	Tetradecanamide	0.06
186	36.72	Ethanol, 2-(9-octadecenyloxy)-, (Z)-	0.02
189	37.13	Capramide, 2,6-diamino-n-hexadecyl-	0.03
190	37.25	Succinic acid, 2-decyl isohexyl ester	0.03
193	37.53	11-Dodecen-1-ol trifluoroacetate	0.01
206	39.45	13-Docosenamide, (Z)-	0.13
212	40.45	Bis(pentamethylcyclopentadienyl)iron	0.08
216	41.4	4-Aminobutyramide, N-methyl-N-[4-(1-pyrrolidinyl)-2-butynyl]-	0.09
229	45.61	Spinasterone	0.02
