# Supplementary material for: Research on using Aquilaria sinensis callus to evaluate the agarwood-inducing potential of fungi
Source: PLoS One. 2024 Dec 26;19(12):e0316178. doi: 10.1371/journal.pone.0316178 (PMC11671001; doi:10.1371/journal.pone.0316178)
Supplement: S2 Table — (PDF) [file pone.0316178.s003.pdf]

S3 Table. GS-MS results of W-15 treatment.

No.	Retention Time (min)	Compound	Relative amount / %
		**Sesquiterpenes**	**16.69**
85	21.16	Benzylideneacetone	0.04
92	22.09	Camphene	0.01
108	24.33	alpha-Curcumene	0.03
121	25.96	Neoisolongifolene, 8,9-dehydro-	0.04
127	26.6	(+)-trans-Isolimonene	0.16
128	26.74	alpha.-Santalol	0.68
132	27.17	Methyl sandaracopimarate	0.06
135	27.52	Eudesma-4(14),7(11)-diene	0.06
136	27.61	beta-Maaliene	0.21
141	28.2	alpha-Elemene	0.53
142	28.26	beta-Selinene	0.55
143	28.33	alpha-Himachalene	0.17
144	28.45	2-Methoxy-4-methyl-bicyclo[3.2.1]oct-2-ene	0.23
146	28.64	Aromadendrene, dehydro-	0.14
153	29.24	o-Mentha-1(7),8-dien-3-ol	0.32
157	29.82	Dehydrofukinone	0.67
160	30.11	Aromadendrane	0.27
162	30.31	Caryophyllene oxide	0.27
164	30.46	Longifolenaldehyde	0.32
168	30.78	Ledene oxide-(I)	0.17
176	31.33	Caryophyllene	0.27
177	31.38	Alloaromadendrene	0.67
179	31.69	γ-Gurjunenepoxide-（2）	2.48
180	31.86	Cedran-diol, 8S,13-	0.25
181	31.89	Caryophyllene-(I1)	0.23
182	31.99	Cycloheptane,1-ethenyl-1-methyl-4-methylene-2-(2-methyl-1-propenyl)-	0.36
186	32.82	gamma-Neoclovene	3.15
193	33.3	Isoaromadendrene epoxide	0.61
194	33.36	Aromadendrene oxide 2	0.72
203	34.48	Farnesyl acetate	0.23
209	34.92	(2Z,4E)-3,7,11-Trimethyl-2,4,10-dodecatriene	0.12
215	35.44	viridiflorene	0.12
217	35.76	Farnesol	0.15
249	39.8	Squalene	2.56
295	47.02	7-epi-cis-sesquisabinene hydrate	0.11
		**Aromatic compounds**	**21.6**

(Continued)

S3 Table. (Continued)

8	4.58	Benzoyl bromide	0.37
34	9.82	Benzaldehyde	0.38
43	12.23	Benzaldehyde, 3-hydroxy-	0.05
47	12.68	2,5-Dimethyl-4-hydroxy-3(2H)-furanone	0.01
48	12.8	3-[2-(3-Methoxy-phenyl)-2-oxo-ethyl]-5-phenyl-3H-[1,3,4]oxadiazol-2-one	0.01
53	13.6	Phenol, 2-methoxy-	0.01
55	14.21	Benzaldehyde, 2,5-bis[(trimethylsilyl)oxy]-	0.01
61	15.75	Ethanone, 1-(2-hydroxyphenyl)-	0.02
62	15.96	Benzoic acid	0.02
63	16.18	Butanedioic acid, diethyl ester	0.01
66	17.3	Phenol, 4-amino-3,5-dichloro-	0.01
68	17.82	5-Hydroxymethylfurfural	0.01
70	18.08	2-Butanone, 4-phenyl-	0.77
71	18.32	Benzene, 1,3-bis(1,1-dimethylethyl)-	0.01
72	18.38	Benzaldehyde, 4-methoxy-	0.04
74	18.79	FENAZAQUIN	0.01
79	19.98	2-Methoxy-4-vinylphenol	0.03
81	20.36	Benzene, 1-methyl-4-(1-methylethenyl)-	0.01
82	20.78	Hydrocinnamic acid	0.40
83	20.89	Benzenepropanoic acid, ethyl ester	0.03
84	20.96	Benzene, 1-methoxy-2-(methylthio)-	0.03
86	21.23	2',6'-Dihydroxyacetophenone, bis(trimethylsilyl) ether	0.02
88	21.57	Benadryl	0.01
93	22.26	Benzaldehyde, 3-hydroxy-4-methoxy-	0.03
94	22.39	Vanillin	0.01
96	22.8	Naphthalene, 2,7-dimethyl-	0.01
102	23.61	Phenol, 2,4,6-tris(1-methylethyl)-	0.03
107	24.23	Butylated Hydroxytoluene	0.14
109	24.48	Ethanone, 1-cyclopropyl-2-phenyl-	0.06
111	24.78	2-Butanone, 4-(4-methoxyphenyl)-	0.87
112	24.92	Benzenamine, 3,5-dimethyl-	0.06
113	25	Phenol, 2,4-bis(1,1-dimethylethyl)-	0.07
116	25.33	Phenol, 4,6-di(1,1-dimethylethyl)-2-methyl-	0.02
120	25.86	Benzimidazole, 2-amino-1-methyl-	0.02
123	26.14	2-Butanone, 4-(4-hydroxyphenyl)-	0.1
124	26.32	4-Methyl-2,5-dimethoxybenzaldehyde	0.1
126	26.5	1-Naphthalenemethanol, 1,2,3,4-tetrahydro-8-methyl-	0.03
129	26.84	3-(4-Methoxyphenyl)propionic acid	0.35
131	27.05	3-(4-Methoxyphenyl)propionic acid ethyl ester	0.05
133	27.28	Carbonic acid, 3,4-dimethylphenyl propargyl ester	0.04

(Continued)

S3 Table. (Continued)

134	27.37	2,3-Dimethylphenoxyacetic acid	0.08
145	28.55	1-Ethyl-2-benzimidazolinone	0.67
147	28.7	Benzenebutanal, .gamma.,4-dimethyl-	0.05
204	34.52	1-Allyldimethylsilyloxy-4-methoxybenzene	0.14
206	34.66	1-Penten-3-one, 1,5-diphenyl-	0.33
223	36.31	8-Naphthol, 1-(benzyloxy)-	2.32
233	37.74	Bis(2-ethylhexyl) phthalate	0.38
234	37.87	4-(2-Furamido)benzoic acid	0.08
236	38.11	2-(Salicylideneamino)naphthalene	0.06
237	38.2	Benzenamine, 2,5-dimethyl-	0.14
238	38.4	9H-Benzo[4,5]imidazo[2,1-c][1,2,4]triazole, 3-benzylsulfanyl-	0.77
239	38.44	4-(4-Methoxyphenyl)-1-butanol	0.43
240	38.51	Butane, 1-(benzyloxy)-2-[(benzyloxy)methyl]-	0.1
242	38.81	Coumarin, 6-benzyloxy-3,4-dihydro-4,4-dimethyl-	1.47
243	38.94	Naphtho[1,8,8a-3',5',4']pyrido[1',2'-a]imidazolo[4'',5''-f]benzimidazol-7,11(10H,12H)-dione, 10,12-dimethyl-	0.11
244	39.01	5-Acetyl-2-benzylsulfanyl-6-methyl-nicotinonitrile	0.06
246	39.22	Acetamide, N-(4-benzyloxyphenyl)-2-cyano-	1.04
248	39.59	(E)-1-(4-Methylphenyl)butan-1-one O-(tert-butyldimethylsilyl)oxime	0.28
250	39.92	2-Methoxybenzyl alcohol, 2-methylbutyl ether	0.55
251	40.09	5,6-Dihydro-4,4,6-trimethyl-2,6-diphenyl-4H-1,3-thiazine	0.43
252	40.23	Pentanoic acid, 3-amino-4-(dibenzylamino)-5-phenyl-, ethyl ester	0.47
253	40.38	5-Fluoro-1,3-bis[phenylmethyl]-2,4(1H,3H)-pyrimidinedione	2.28
254	40.51	1,2,3,4-Tetrahydroisoquinoline, N-acetyl-6-benzyloxy-7-methoxy-1-methyl	0.07
256	40.72	Benzene, 1-trifluoromethyl-4-(3-methoxybenzyloxy)-3-nitro-	0.15
257	40.77	Quinazolin-4(3H)-one, 2-(4-methoxybenzylthio)-3-methyl-	1.31
259	41.04	Indolo[3,2-b]indole, 5-acetyl-10-benzyl-5,10-dihydro-	0.1
260	41.41	Glutaric acid, 2-methoxybenzyl propyl ester	0.15
263	41.7	2-Methoxybenzyl alcohol, 3-methylbutyl ether	0.1
269	42.34	Butylphosphonic acid, isobutyl 4-methoxybenzyl ester	0.6
270	42.51	Bibenzyl, 4,4'-dimethoxy-	1.33
271	42.63	1H-[1,2,3]Triazole-4-carboxamide, 5-amino-N-(4-fluorophenyl)-1-(4-methoxybenzyl)-	0.16
272	42.72	Benzene, 1-methoxy-3-(3-methyl-3-butenyl)-	0.07
273	42.77	(4-Methoxy-benzyl)-phenethyl-amine	0.1
276	43.1	Homovanillyl alcohol	0.14
277	43.36	p-(Pentyloxy)acetophenone	0.28
278	43.43	4-Methoxy-.alpha.-toluenethiol	0.09

(Continued)

S3 Table. (Continued)

279	43.53	4-Hydroxybenzoic acid, 3,10,10-trimethyl-7-methylenecycloundeca-3,8-dienyl ester	0.09
280	43.62	Pyrido[1,2-a][1,3]benzimidazole-4-carbonitrile, 1-[[(3-methoxyphenyl)methyl]amino]-3-methyl-	0.1
285	45.1	1-(4-Methoxybenzyloxy)-1-methyl-1-silacyclopentane	0.11
289	46.3	Benzhydrazide, 2-hexyloxy-N2-(2,3-dihydro-2-oxoindol-3-ylideno)-	0.04
294	46.91	Phenol, 2-[3-(trifluoromethyl)-5-isoxazolyl]-	0.02
299	48.07	Ethyl 2-butyramido-3,3,3-trifluoro-2-(4-fluoroanilino)propionate	0.01
300	48.26	Butylphosphonic acid, 4-formylphenyl hexyl ester	0.04
302	48.74	2-Methyl-1-(3,4-dimethoxyphenyl)-1-(2-methylamino-4,5-dimethoxyphenyl)propane	0.04
		**Chromones**	**3.17**
49	12.91	Acetophenone	0.02
91	21.9	Hydrocoumarin	0.04
159	30.04	2(1H)Naphthalenone, 3,5,6,7,8,8a-hexahydro-4,8a-dimethyl-6-(1-methylethenyl)-	0.42
187	32.9	2H-Cyclopropa[a]naphthalen-2-one,1,1a,4,5,6,7,7a,7b-octahydro-1,1,7,7a-tetramethyl-, (1a.alpha.,7.alpha.,7a.alpha.,7b.alpha.)-	1.08
199	33.87	1,4-Naphthalenedione, 2-hydroxy-3-propyl-	0.38
200	34.01	2(1H)-Naphthalenone, 4a,5,6,7,8,8a-hexahydro-7.alpha.-isopropyl-4a.beta.,8a.beta.-dimethyl-	0.2
245	39.09	Methylchromone	0.13
258	40.94	Norgestrel	0.45
262	41.54	Ethanone, 1-[5-(2-furanylmethyl)-2-furanyl]-	0.18
274	42.88	1-(2-Ethoxyphenyl)-5-(4-methoxybenzyl)-2,4,6(1H,3H,5H)-pyrimidinetrione	0.06
287	45.63	2-Oxazolidinone, 4-phenyl-5-p-tolyl-, trans-	0.09
291	46.5	Stigmast-4-en-3-one	0.04
303	49.01	Chol-9(11)-en-12-one, 3-hydroxy-, (3.alpha.,5.beta.)-	0.07
305	49.66	12H-[1,3]Benzoxazolo[2,3-b]quinazolin-12-one	0.01
		**Alkanes**	**56.6**
1	3.23	Silanol, trimethyl-	0.55
2	3.45	Ethyl Acetate	1.91
3	3.75	Acetic acid	2.97
4	3.89	2-Propanone, 1-hydroxy-	0.03
5	4.13	N,N-Dimethylformamide dipropyl acetal	0.02
6	4.33	Acetoin	0.28
7	4.39	Propane, 1-(1-ethoxyethoxy)-	0.09
9	4.72	1,3-Dioxan-5-ol	0.01

(Continued)

S3 Table. (Continued)

10	4.95	Propanamide, N,N-dimethyl-	0.09
11	5.11	Silane, diethoxydimethyl-	0.02
12	5.33	Ethanol, 2-methoxy-	0.01
13	5.55	2,3-Butanediol	3.05
14	5.96	2,3-Butanediol, [R-(R*,R*)]-	4
15	6.17	Ethane, 1,1-diethoxy-	0.02
16	6.26	Butane, 2-ethoxy-	1.11
17	6.45	1H-Indole, 1-methyl-	0.04
18	6.54	Furfural	0.06
19	7.04	3-Furanmethanol	0.01
20	7.27	Acetic acid, methoxy-, methyl ester	0.13
21	7.47	1,3-Butanediol, (S)-	0.01
22	7.53	Butane, 1,4-bis(5-thioxo-1,2,4-triazol-2-in-3-yl)-	0.02
23	7.82	CH2=C(CH3)CH2COOH	0.13
24	7.91	CH3C(O)OCH(CH3)C(O)CH3	0.02
25	8.11	2,2,6-Trimethyl-4H-1,3-dioxin-4-one	0.04
26	8.16	2-Butenoic acid, 3-methyl-	0.01
27	8.23	Acetic acid, dimethoxy-, methyl ester	0.01
28	8.29	1-Propanol, 3-ethoxy-	0.01
29	8.49	Butyrolactone	0.02
30	8.74	Oxirane, trimethyl-	0.01
31	9.07	Acetic acid, methoxy-, ethyl ester	0.08
32	9.44	Ethyl acetoacetate	0.08
33	9.59	m-Tolualdehyde, thiosemicarbazone	0.01
35	9.91	Benzeneacetic acid, tert-butyldimethylsilyl ester	0.01
36	10.45	Phenol	0.02
37	10.59	Ethanedioic acid, diethyl ester	0.01
38	10.87	Glutaric acid, di(5-methoxy-3-methylpent-2-yl) ester	0.01
39	11.45	2-Hexanol, 3-methyl-	0.02
40	11.53	Hydrazine, (4-methylphenyl)-	0.02
41	11.74	1-Hexanol, 2-ethyl-	0.03
42	12.04	2(3H)-Furanone, dihydro-3-hydroxy-4,4-dimethyl-, (.+/-.)-	0.01
44	12.29	tert-Butyldimethylsilyl formate	0.01
45	12.49	2-Butene, 2,3-dimethyl-	0.01
46	12.55	Trifluoromethylthiocyanate	0.01
50	12.98	Propanedioic acid, diethyl ester	0.01
51	13.22	Butyraldehyde, semicarbazone	0.03
52	13.48	1,3-Dimethyl-3,4,5,6-tetrahydro-2(1H)-pyrimidinone	0.01
54	13.74	2-Propanamine	0.02
56	14.74	Thiazole	0.01
57	14.83	2-Hexene, 1-(1-ethoxyethoxy)-, (E)-	0.01

(Continued)

S3 Table. (Continued)

58	15.26	1-Propene-1-thiol	0.04
59	15.37	Galactitol	0.02
60	15.55	trans-2,3-Epoxynonane	0.04
64	16.75	Cyclopentane, 1,3-dimethyl-	0.02
65	17.08	Silane, dimethyl(dimethyl(dodec-2-enyloxy)silyloxy)ethoxy-	0.05
67	17.36	Benzofuran, 2,3-dihydro-	0.01
69	17.9	Thiophene, 2-propyl-	0.01
73	18.66	2-Undecenal	0.12
75	19.2	1-Nonene	0.01
76	19.31	3-Piperidinone, 1-ethyl-	0.04
77	19.48	Naphthalene, 1-methyl-	0.01
78	19.79	Piperidine, 1-acetyl-	0.02
80	20.08	Phthalic acid, monoethyl ester	0.01
87	21.38	Disparlure	0.03
89	21.65	1-Heptene, 5-methyl-	0.01
90	21.79	Chloroacetic acid, 4-methoxy-2-methylbutyl ester	0.01
95	22.69	14-Methylhexadec-9-enoic acid, methyl ester	0.15
97	22.97	1,4-Butanediamine, N'-[4-(dimethylamino)butyl]-N,N-dimethyl-	0.02
98	23.06	N,N-Dimethyl-3-ethoxypropylamine	0.01
99	23.18	Acetic acid, chloro-, pentyl ester	0.01
100	23.3	Pentane, 1-bromo-5-chloro-	0.01
101	23.53	Cyclopentane, butyl-	0.03
103	23.73	1,4,7,-Cycloundecatriene, 1,5,9,9-tetramethyl-, Z,Z,Z-	0.08
104	23.89	Methyl N-methyl-N-formyldithiocarbamate	0.05
105	23.95	1,2-Benzenedicarboxylic acid, diundecyl ester	0.03
106	24.11	Tridecanal	0.04
110	24.61	cis-2,6-Dimethyl-2,6-octadiene	0.02
114	25.09	Cyclohexane, 1R-acetamido-2cis,4trans-bis(acetoxy)-3trans-azido-	0.02
115	25.21	2H-1,2-Oxazine, tetrahydro-2-methyl-6-(3-pyridinyl)-, (-)-	0.01
117	25.42	4H-1-Benzopyran-4-one, 2-methyl-	0.12
118	25.67	Dodecanoic acid	0.01
119	25.77	2-Cyclohexen-1-ol	0.02
122	26.01	Bicylo[4.1.0]heptane, 7-bicyclo[4.1.0]hept-7-ylidene-	0.03
125	26.46	5-Benzothiazolamine, 2-methyl-	0.03
130	26.97	exo-Bicyclo[3.3.0]octane-2-carboxylic acid, 4-methylene-, methyl ester, cis--	0.06
137	27.79	.gama.-eudesmol	0.08
138	27.88	4-epi-cubedol	0.22
139	27.97	2,10,10-Trimethyltricyclo[7.1.1.0(2,7)]undec-7-en-6-one	0.21

(Continued)

S3 Table. (Continued)

140	28.05	Tricyclo[4.1.0.0(2,4)]heptane, 3,3,7,7-tetramethyl-5-(2-methyl-1-propenyl)-	0.42
148	28.76	Phosphinic acid, dipropyl-, propyl ester	0.06
149	28.83	4,6,6-Trimethyl-2-(3-methylbuta-1,3-dienyl)-3-oxatricyclo[5.1.0.0(2,4)]octane	0.76
150	28.89	1-Formyl-2,2,6-trimethyl-3-(3-methyl-but-2-enyl)-6-cyclohexene	0.29
151	28.98	Benzene, 1-cyclohexyl-2-methoxy-	0.75
152	29.07	1,5,9,11-Tridecatetraene, 12-methyl-, (E,E)-	0.26
154	29.42	6,6,10-Trimethylundeca-3,8,10-triene-2,7-dione	0.69
155	29.56	3,6-Nonadien-5-one, 2,2,8,8-tetramethyl-	0.14
156	29.68	Tetradecanoic acid	0.46
158	29.92	(+)-3-Carene, 2-.alpha.-isopropenyl-	0.36
161	30.23	7-Oxabicyclo[4.1.0]heptane, 2,2,6-trimethyl-1-(3-methyl-1,3-butadienyl)-5-methylene-	0.16
163	30.39	Glaucyl alcohol	0.16
165	30.52	1H-Indene, 1-ethylideneoctahydro-7a-methyl-, (1Z,3a.alpha.,7a.beta.)-	0.28
166	30.61	2-(1-Cyclohexenyl)cyclohexanone	0.78
167	30.7	But-3-enal, 2-methyl-4-(2,6,6-trimethyl-1-cyclohexenyl)-	0.38
169	30.81	1-Methylene-2b-hydroxymethyl-3,3-dimethyl-4b-(3-methylbut-2-enyl)-cyclohexane	0.16
170	30.88	Cyclobutane, tetrakis(1-methylethylidene)-	0.27
171	30.98	Hydrazine, (3-fluorophenyl)-	0.56
172	31.08	1,3-Benzenedicarboxylic acid, 4-methyl-, dimethyl ester	2.28
173	31.15	2-Guanidino-4(3H)-quinazolinone	0.87
174	31.24	9-Borabicyclo[3.3.1]nonane, 9-[(4-pyridyl)amino]-	0.28
175	31.27	Tricyclo[4.3.1.1(3,8)]undecane, 1-chloro-	0.22
178	31.53	(3S,4R,5R,6R)-4,5-Bis(hydroxymethyl)-3,6-dimethylcyclohexene	0.45
183	32.19	4-Tridecen-6-yne, (E)-	2.95
184	32.42	n-Hexadecanoic acid	3.23
185	32.61	4,4'-Dimethyl-2,2'-dimethylenebicyclohexyl-3,3'-diene	3.5
188	32.98	1H-Pyrrole, 1-butyl-	0.39
189	33.02	Allyl(2-pentyloxy)dimethylsilane	0.2
190	33.11	2,2,6-Trimethyl-1-(2-methyl-cyclobut-2-enyl)-hepta-4,6-dien-3-one	0.53
191	33.2	.gamma.-Gurjunenepoxide-(1)	0.79
192	33.25	(-)-Isolongifolol, methyl ether	0.35
195	33.5	6-(1-Hydroxymethylvinyl)-4,8a-dimethyl-3,5,6,7,8,8a-hexahydro-1H-naphthalen-2-one	3.09

(Continued)

S3 Table. (Continued)

196	33.59	Naphthalene, 1,2,3,4-tetrahydro-6-nitro-	0.38
197	33.7	Silane, trimethyl(2-naphthalenyloxy)-	2.14
198	33.79	Pyridazine, 3-(1,3-benzodioxol-5-yl)-6-hydrazino-	1.33
201	34.24	Oleic Acid	2.58
202	34.41	Octadecanoic acid	0.83
205	34.61	Hexadecanamide	0.21
207	34.74	Z,Z-3,15-Octadecadien-1-ol acetate	0.07
208	34.84	Tetrazolo[1,5-c]quinozalin-5(6H)-one	0.13
210	34.96	Cyclohexene, 4-(4-ethylcyclohexyl)-1-pentyl-	0.16
211	35.06	Alloaromadendrene oxide-(1)	0.32
212	35.25	Butanedioic acid, decyl phenylmethyl ester	0.11
213	35.3	1,2-Benzisothiazole, 3-(hexahydro-1H-azepin-1-yl)-, 1,1-dioxide	0.16
214	35.39	4,4-Dimethyl-3-(3-methylbut-3-enylidene)-2-methylenebicyclo[4.1.0]heptane	0.07
216	35.51	Cyclohexadecane, 1,2-diethyl-	0.21
218	35.83	Ledene oxide-(II)	0.24
219	35.95	Cyclohexaneethanol, 4-methyl-.beta.-methylene-	0.13
220	36.02	1-Eicosene	0.11
221	36.09	2-Dodecen-1-yl(-)succinic anhydride	0.05
222	36.17	Cycloeicosane	0.1
224	36.44	Tetracosane	0.21
225	36.62	Diacetyldihydrofluoresceyl dihydroqinghaosu	0.06
226	36.72	E,E,Z-1,3,12-Nonadecatriene-5,14-diol	0.07
227	36.93	Chloroacetic acid, pentadecyl ester	0.06
228	36.99	5-Octadecene, (E)-	0.05
229	37.04	9-Eicosene, (E)-	0.07
230	37.13	1-Heptadecene	0.07
231	37.28	Trifluoroacetic acid,n-tridecyl ester	0.27
232	37.42	1-Docosene	0.08
235	37.98	2-(E)-Hexen-1-ol, 4-(dibenzylamino)-5-methyl-	0.42
241	38.63	Fumaric acid, but-3-yn-2-yl dodecyl ester	0.07
247	39.46	13-Docosenamide, (Z)-	0.57
255	40.55	4,4'-Dipentylazoxybenzene	0.06
261	41.47	Salicylhydrazide, N2-(2,4-dichlorobenzylideno)-	0.04
264	41.79	17-Pentatriacontene	0.1
265	41.84	22-Tricosenoic acid	0.08
266	41.9	Oxacyclotetradecan-2-one, 14-methyl-	0.06
267	42.03	Silane,dimethyl(trans-3,7-dimethyl-2,6-octadien-1-yloxy)dodecyloxy-	0.23
268	42.18	9-Hexadecenoic acid, eicosyl ester, (Z)-	0.17

(Continued)

S3 Table. (Continued)

275	42.97	2,4,6,8,9,10-Hexaaza-1,3,5,7-tetraphosphatricyclo[3.3.1.1(3,7)]decane,2,4,6,8,9,10-hexamethyl-, 1-sulfide	0.09
281	43.88	Stigmasterol	0.37
282	44.29	Cyclotriacontane	0.12
283	44.58	.gamma.-Sitosterol	0.32
284	44.78	5-Chlorovaleric acid, octadecyl ester	0.22
286	45.28	1-Octadecene	0.1
288	46.02	Glutaric acid, 2-methoxybenzyl undecyl ester	0.11
290	46.38	Cyclopropaneoctanoic acid, 2-hexyl-, methyl ester	0.03
292	46.6	Cyclopropanedecanoic acid, 2-hexyl-, methyl ester	0.06
293	46.73	3-Eicosene, (E)-	0.03
296	47.36	1-Nonadecene	0.01
297	47.52	Hexadecanoic acid, octadecyl ester	0.04
298	47.91	9-Tricosene, (Z)-	0.02
301	48.62	cis-10-Nonadecenoic acid	0.02
304	49.29	Cyclohexanecarboxamide, N-furfuryl-	0.01
