# Supplementary material for: Research on using Aquilaria sinensis callus to evaluate the agarwood-inducing potential of fungi
Source: PLoS One. 2024 Dec 26;19(12):e0316178. doi: 10.1371/journal.pone.0316178 (PMC11671001; doi:10.1371/journal.pone.0316178)
Supplement: S3 Table — (PDF) [file pone.0316178.s004.pdf]

S4 Table. GS-MS results of YMY treatment.

No.	Retention Time (min)	Compound	Relative amount / %
**Sesquiterpenes**			**10.12**
84	26.01	Neoisolongifolene, 8,9-dehydro-	0.02
88	26.61	4-methyl-1-prop-1-en-2-ylcyclohexene	0.26
95	27.61	beta-Maaliene	0.14
97	27.79	(-)-alpha-Gurjunene	0.01
98	27.87	Hinesol	0.08
99	27.94	(+)-Eremophilene	0.06
100	28.05	Longifolene	0.17
101	28.19	Bicyclo[4.4.0]dec-1-ene, 2-isopropyl-5-methyl-9-methylene-	0.29
102	28.24	gamma-Gurjunene	0.23
103	28.33	(-)-Globulol	0.14
106	28.63	Cycloisolongifolene, 8,9-dehydro-	0.08
108	28.87	1,8-dimethyl-4-(1-methylethyl)-spiro[4.5]dec-8-en-7-one	0.28
112	29.24	Culmorin	0.15
125	30.62	Dehydrofukinone	0.35
126	30.71	beta-EUDESMOL	0.12
127	30.75	Cycloheptane, 1-ethenyl-1-methyl-4-methylene-2-(2-methyl-1-propenyl)-	1.89
128	30.86	Alpha-Farnesene	0.11
131	31.13	cis-Thujopsene	0.35
132	31.25	Z-beta-Guaiene	0.38
136	31.5	gamma-Elemene	0.19
137	31.65	gamma-Gurjunenepoxide-(2)	0.85
138	31.71	Alloaromadendrene	1.41
141	31.99	Guaia-1(10),11-diene	0.32
152	33.22	4a,5-dimethyl-3-prop-1-en-2-yl-2,3,4,5,6,7-hexahydro-1H-naphthalene	0.24
153	33.32	Aromandendrene	0.37
155	33.56	beta-Ionone	0.34
158	33.75	Procerin	0.36
166	34.55	Aromadendrene oxide-(2)	0.08
170	34.85	beta-Apo-13-carotenone	0.06
176	35.41	Limonene dioxide	0.05
215	39.77	Squalene	0.23
220	40.45	Ajmaline	0.08
238	45.12	Methyl isodextropimarate	0.43
**Aromatic compounds**			**16.66**

(Continued)

S4 Table. (Continued)

20	7.93	Styrene	0.01
26	9.85	Benzaldehyde	0.19
39	12.25	Benzaldehyde, 2-hydroxy-	0.01
56	17.61	Ethanone, 2-ethoxy-1,2-diphenyl-	0.13
57	18.14	2-Butanone, 4-phenyl-	2.22
60	18.42	Benzaldehyde, 3-methoxy-	0.04
64	20.69	Hydrocinnamic acid	0.2
65	20.91	Benzenepropanoic acid, ethyl ester	0.03
68	21.2	3-Buten-2-one, 4-phenyl-	0.01
80	25.33	Butylated Hydroxytoluene	0.01
83	25.91	Benzene, 1-(1,1-dimethylethyl)-3-ethyl-	0.03
85	26.14	Fenipentol	0.02
92	27.29	Isopropyl phenyl ketone	0.01
113	29.41	Acetic acid, trifluoro-, 2-methoxyphenyl ester	0.34
129	30.95	Benzene, 1-fluoro-4-methoxy-	0.36
134	31.36	Adipic acid, diphenyl ester	0.15
143	32.4	Dibutyl phthalate	0.24
151	33.15	4,5-Diphenylocta-1,7-diene(meso)	0.3
156	33.65	Cyclohexanone, 3,3,5-trimethyl-5-phenyl-	0.61
159	33.82	Spiro[2,3-dihydroindol-3,5'-2'-thiazoline],2'-methylthio-2-oxo-	0.16
168	34.64	1-Penten-3-one, 1,5-diphenyl-	0.23
169	34.77	Butanedioic acid, heptyl phenylmethyl ester	0.15
175	35.33	2,5-Difluorobenzyl alcohol, 1-methylpropyl ether	0.32
177	35.5	Urea, N-(2-chlorophenyl)-N'-phenyl-	0.09
183	36.02	Levulinic acid, 5-phenyl-, ethyl ester	0.1
184	36.1	Benzene, 1,4-diethyl-2,3,5,6-tetramethyl-	0.03
185	36.16	Butanedioic acid, butyl phenylmethyl ester	0.04
186	36.3	8-Naphthol, 1-(benzyloxy)-	2.66
192	36.8	2-(E)-Penten-1-ol, 5-(benzyloxy)-4-(dibenzylamino)-5-phenyl-	0.12
197	37.38	Pentanamine, 2-(dibenzylamino)-4-methyl-	0.05
199	37.57	Butanedioic acid, diethyl ester	0.02
201	37.85	1,3-Dioxolane, 5-benzyloxymethyl-2,2-dimethyl-4-formyl-(5R)-	0.01
203	38.11	Pyridine, 3-((o-chlorophenyl)azo)-2,6-diamino-	0.04
204	38.19	6-[4-Methoxybenzyloxy]-8-nitrolepidine	0.06
206	38.43	Benzene, 1-methoxy-4-undecyl-	0.46
209	38.8	Coumarin, 6-benzyloxy-3,4-dihydro-4,4-dimethyl-	0.68
211	39.21	Acetamide, N-(4-benzyloxyphenyl)-2-cyano-	1.19
213	39.58	(S)-(-)-1-Benzyl-2-pyrrolidinemethanol	0.07

(Continued)

S4 Table. (Continued)

217	40.08	2-[p-Methoxybenzyloxy]-6-methoxy-8-aminoquinoline	0.28
218	40.2	1H-Imidazo[1,2-a]pyridin-4-ium, 3-(chloroacetyl)-2-hydroxy-1-(phenylmethyl)-, hydroxide, inner salt	0.25
219	40.36	5-Fluoro-1,3-bis[phenylmethyl]-2,4(1H,3H)-pyrimidinedione	1.53
221	40.63	Quinazolin-4(3H)-one, 2-(4-methoxybenzylthio)-3-methyl-	0.59
222	40.92	Imidazolidin-4-one, 5-benzyl-2-thioxo-3-p-tolyl-	0.27
223	41.17	4-Methoxybenzylamine, N,N-dihexyl-	0.8
224	41.51	1-Di(t-butyl)silyloxymethyl-4-methoxybenzene	0.1
225	41.67	Phenol, 2-(1-methylpropyl)-	0.08
226	41.79	(4-Methoxy-benzyl)-phenethyl-amine	0.08
228	42.12	1H-[1,2,3]Triazole-4-carboxamide, 5-amino-N-(4-fluorophenyl)-1-(4-methoxybenzyl)-	0.1
229	42.37	Benzimidazole, 5-(4-methoxybenzylamino)-1-phenyl-	0.1
230	42.49	4-Hexylanisole	0.5
231	43.22	Benzene, 1-methoxy-4-pentyl-	0.11
232	43.34	Bibenzyl, 4,4'-dimethoxy-	0.15
233	43.54	[2-(3,4-Dimethoxyphenyl)ethyl](4-methoxybenzyl)amine	0.08
234	43.88	Benzamide, 2-hydroxy-N-[2-(1-methylethoxy)phenyl]-	0.1
237	44.78	2-Hydroxy-2-phenylacetic acid, p-methoxybenzyl ester	0.02
239	45.62	1H-Indole-3-carboxylic acid, 5-hydroxy-	0.01
240	46.35	3,4-Dimethoxybenzylideneacetone	0.12
		**Chromones**	4.38
45	14.26	5,8-Dimethoxycumarin	0.12
69	21.93	Hydrocoumarin	0.01
78	24.84	2-Butanone, 4-(4-methoxyphenyl)-	3.47
81	25.45	4H-1-Benzopyran-4-one, 2-methyl-	0.04
140	31.85	11-Oxatetracyclo[5.3.2.0(2,7).0(2,8)]dodecan-9-one	0.21
145	32.65	6-(1-Hydroxymethylvinyl)-4,8a-dimethyl-3,5,6,7,8,8a-hexahydro-1H-naphthalen-2-one	0.27
216	39.91	Norgestrel	0.25
241	46.49	Stigmast-4-en-3-one	0.01
		**Alkanes**	50.33
1	3.42	Ethyl Acetate	2.05
2	3.98	Acetic acid	4.54
3	4.16	Boronic acid, ethyl-, diethyl ester	0.05
4	4.4	2-Butanol, 3-methyl-, (S)-	2.56
5	4.55	1,3-Dioxolane, 2,4,5-trimethyl-	2.48
6	4.63	Phenacyl thiocyanate	0.53
7	4.77	Propanamide, N,N-dimethyl-	2.36
8	5.15	Silane, diethoxydimethyl-	0.11

(Continued)

S4 Table. (Continued)

9	5.23	2-Oxopentanedioic acid	0.04
10	5.51	N-(2-Methoxyethyl)-N-ethylnitrosamine	1.12
11	5.67	2,3-Butanediol	1.94
12	6.07	2,3-Butanediol, [R-(R*,R*)]-	9.65
13	6.52	Diethyl 2,2'-(2,2'-oxybis(ethane-2,1-diyl)bis(oxy))diacetate	0.19
14	6.8	Silane, [(dimethylsilyl)methyl]trimethyl-	0.05
15	6.86	Silane, butoxytrimethyl-	0.05
16	7.17	1-Propanol, 2-(1-methylethoxy)-	0.01
17	7.44	Acetic acid, methoxy-, methyl ester	1.04
18	7.62	R-(-)-1,2-propanediol	0.01
19	7.76	1,3-Butanediol, (S)-	0.18
21	7.99	CH3C(O)OCH(CH3)C(O)CH3	0.06
22	8.39	Isoxazolidine	0.04
23	8.56	2-Ethoxy-3-chlorobutane	0.02
24	8.98	Pentaethylene glycol	0.32
25	9.16	Acetic acid, methoxy-, ethyl ester	0.52
27	9.93	Pyrazolo[1,5-a]pyrimidine, 7-ethyl-5-methyl-2-phenyl-	0.01
28	10.11	Propane, 2-ethoxy-	0.02
29	10.63	1-Methylpropylhydroxylamine	0.01
30	10.9	Butanamide, N-formyl-2-hydroxy-3-methyl-2-(1-methylethyl)-	0.03
31	11.15	Methoxyacetic acid, 2-pentyl ester	0.02
32	11.39	4-Methoxymethoxy-3-nitro-pentan-2-ol	0.01
33	11.47	2-Methylbutane-1,4-diol, 3-(1-ethoxyethoxy)-	0.01
34	11.55	Hydrazinecarbodithioic acid, methyl ester	0.01
35	11.71	1-Hexanethiol	0.01
36	11.79	2-Dodecanol	0.03
37	11.99	2-Hexen-1-ol, 5-[[(1,1-dimethylethyl)dimethylsilyl]oxy]-, (E)-(.+-.)-	0.01
38	12.08	Ethyl(dimethyl)ethoxysilane	0.08
40	12.32	tert-Butyl-(2-ethoxyethoxy)dimethylsilane	0.11
41	12.94	6-Benzoylhexanoic acid	0.01
42	13.01	Propanedioic acid, diethyl ester	0.01
43	13.07	Butanoic acid, 3-hydroxy-, methyl ester, (S)-	0.01
44	13.98	Butane, 2-ethoxy-	0.01
46	14.3	Phenol, 3-ethyl-5-methyl-	0.01
47	14.88	1,3-Dioxolane, 2-(1-methylethyl)-	0.02
48	14.99	3-(1-Ethoxy-ethoxy)-butan-1-ol	0.02
49	15.3	2-[2-[2-Methoxyethoxy]ethoxy-1,3-dioxalane	0.04
50	15.41	1-Propanol, 2-methyl-2-nitro-	0.05
51	15.56	1,3-Dioxolane, 2-ethyl-	0.01

(Continued)

S4 Table. (Continued)

52	15.72	Boronic acid, ethyl-, dimethyl ester	0.01
53	15.8	Propanoic acid, 2-(1-ethoxyethoxy)-, ethyl ester	0.01
54	15.98	Silane, diethylmethyl-	0.02
55	17.09	Silane, dimethyl(dimethyl(undec-2-enyloxy)silyloxy)ethoxy-	0.03
58	18.24	Phenol, p-(2-methylallyl)-	0.01
59	18.33	p-Pentylacetophenone	0.03
61	19.53	3-Methyl-3-pentanol, trimethylsilyl ether	0.01
62	20.11	1,2-Benzenedicarboxylic acid	0.01
63	20.36	Benzenamine, 2-(1-methylcyclopropyl)-	0.01
66	20.98	2H-Indazol-3-amine, 2-methyl-	0.01
67	21.03	N-Benzylcyclopropanecarboxamide	0.01
70	22.52	Spiro[2.4]heptane, 1,2,4,5-tetramethyl-6-methylene-	0.2
71	22.97	1,3-Dithiane, 2-(tetrahydrofuran-2-on-5-yl)-	0.06
72	23.16	3-(2-Methylphenyl)propionic acid	0.05
73	23.88	Diethyl Phthalate	0.01
74	23.96	5-Cyano-3-ethoxycarbonyl-1,2,3,4-tetrahydro-4,6-dimethyl-2-oxopyridine	0.01
75	24.23	Ethanone, 1-(5,6,7,8-tetrahydro-2,8,8-trimethyl-4H-cyclohepta[b]furan-5-yl)-	0.06
76	24.44	Benzene, 1-methoxy-4-(1-methylethyl)-	0.01
77	24.49	Glutaric acid, pentyl 1-phenylpropyl ester	0.05
79	25	Phenol, 2,4-bis(1,1-dimethylethyl)-	0.18
82	25.68	Phenol, 4,6-di(1,1-dimethylethyl)-2-methyl-	0.01
86	26.34	Disulfide, diphenyl	0.05
87	26.46	6-Nitro-1H-quinazoline-2,4-dione	0.2
89	26.72	3-(4-Methoxyphenyl)propionic acid	0.26
90	26.97	2-Buten-1-one, 1-(4-hydroxyphenyl)-	0.04
91	27.1	1,1,4a-Trimethyl-5,6-dimethylenedecahydronaphthalene	0.03
93	27.39	3-Cyclohexen-1-carboxaldehyde, 3,4-dimethyl-	0.01
94	27.51	Bicyclo[2.1.1]hexane-5-carboxylic acid, 5-methoxy-3-methylene-, methyl ester	0.02
96	27.73	2-Propanone, 1-phenylthio-3-chloro-	0.02
104	28.46	1,2,4,4-Tetramethylcyclopentene	0.12
105	28.54	Butan-2-one, 4-(3-hydroxy-2-methoxyphenyl)-	0.35
107	28.75	Bicyclo[4.4.0]dec-2-ene-4-ol, 2-methyl-9-(prop-1-en-3-ol-2-yl)-	0.04
109	28.96	1H-Pyrazole-4-carboxaldehyde, 1-(4-fluorophenyl)-	0.31
110	29.05	Tetracyclo[6.3.1.0(2.6).1(6.10)]tridecane	0.09
111	29.1	Cyclohexanone, 2,6-bis(2-methylpropylidene)-	0.06
114	29.53	4-Chlorobenzenesulfonamide, N-methyl-	0.08

(Continued)

S4 Table. (Continued)

115	29.63	2,3-Dimethylphenol, trifluoroacetate	0.19
116	29.73	Tetradecanoic acid	0.07
117	29.81	9H-Cycloisolongifolene, 8-oxo-	0.35
118	29.86	Phenol, 2-ethyl-4,5-dimethyl-	0.22
119	29.91	Bicyclo[5.2.0]nonane, 4-methylene-2,8,8-trimethyl-2-vinyl-	0.29
120	30.03	2(1H)Naphthalenone, 3,5,6,7,8,8a-hexahydro-4,8a-dimethyl-6-(1-methylethenyl)-	0.15
121	30.21	1-(3-Methyl-cyclopent-2-enyl)-cyclohexene	0.19
122	30.29	(-)-Isolongifolol, methyl ether	0.21
123	30.36	4-Pyridinol-1-oxide	0.17
124	30.5	1H-Indene, 1-ethylideneoctahydro-7a-methyl-, (1Z,3a.alpha.,7a.beta.)-	0.23
130	31.05	7-Isobutoxy-5,9-dihydro-6,8-dioxa-7-bora-benzocycloheptene	1.63
133	31.3	6-Isopropenyl-4,8a-dimethyl-4a,5,6,7,8,8a-hexahydro-1H-naphthalen-2-one	0.17
135	31.43	(-)-Isoaromadendrene-(V)	0.15
139	31.8	2-Naphthalenecarboxylic acid, 8-ethenyl-3,4,4a,5,6,7,8,8a-octahydro-5-methylene-	0.08
142	32.35	n-Hexadecanoic acid	1.45
144	32.57	Pyrazole, 1,3,5-trimethyl-4-(3,5-dimethyl-4-pyrazolylazo)-	1.64
146	32.77	Thiourea, 1-(2,4,6-trimethylphenyl)-3-(2-propynyl)-	1.61
147	32.84	Bicyclo[4.1.0]heptan-2-one, 3,4,4-trimethyl-3-(3-methyl-1,3-butadienyl)-, [1.alpha.,3.alpha.(E),6.alpha.]-(.+-.)-	0.47
148	32.91	N-(2,6-Diethylphenyl)-2,2,3,3,3-pentafluoropropanamide	0.36
149	33.01	3-Benzyl-[1,2]dithiolane	0.16
150	33.06	3,6-Nonadien-5-one, 2,2,8,8-tetramethyl-	0.21
154	33.51	5-Aminomethylene-6-hydroxy-4-methyl-2-oxo-2,5-dihydro-3-pyridinecarbonitrile	0.31
157	33.7	2-Methyl-5-nitro-2H-indazole	0.36
160	33.93	3,5,1,7-[1,2,3,4]Butanetetraylnaphthalene-1,6(2H)-diol, octahydro-	0.11
161	33.97	1,4-Methano-1H-indene, octahydro-1,7a-dimethyl-4-(1-methylethenyl)-, [1S-(1.alpha.,3a.beta.,4.alpha.,7a.beta.)]-	0.09
162	34.08	Tricyclo[5.2.2.0(1,6)]undecan-3-ol, 2-methylene-6,8,8-trimethyl-	0.24
163	34.17	Oleic Acid	0.74
164	34.37	Octadecanoic acid	0.81
165	34.46	1,5-Dithiaspiro[5.5]undecane, 10-methyl-7-(1-methylethyl)-,1-oxide,[6R-[6.alpha.(R*),7.beta.,10.alpha.]]-	0.26
167	34.59	Hexadecanamide	0.14

(Continued)

S4 Table. (Continued)

171	34.92	Succinic acid, 3,7-dimethyloct-6-en-1-yl propyl ester	0.2
172	35.03	Heptyl (E)-2-methylbut-2-enoate	0.18
173	35.15	Succinic acid, 3-heptyl nonyl ester	0.41
174	35.25	2-Amino-4-ethyl-5-methyl-thiophene-3-carboxylic acid methyl ester	0.27
178	35.54	Succinic acid, butyl 2,7-dimethyloctnon-5-yn-7-en-4-yl ester	0.05
179	35.6	Succinic acid, ethyl 3-ethylphenyl ester	0.06
180	35.7	Succinic acid, butyl tetradecyl ester	0.17
181	35.76	1,5-Anhydro-2,3,4,6-tetra-O-methyl-D-mannitol	0.08
182	35.82	2-Formyl-4-methylpentanoic acid, ethyl ester	0.21
187	36.41	Octadecanamide	0.15
188	36.56	Succinic acid, hexadecyl 2,2,2-trichloroethyl ester	0.03
189	36.63	1-Butanol, 3-benzyloxy-2-methyl-1-(2,2-dimethyldioxolan-4-yl)-, methanesulfonate	0.02
190	36.67	3-Isopropylimidazolidine-2,4-dione	0.03
191	36.72	Acetoxyacetic acid, 4-hexadecyl ester	0.04
193	36.96	3-S-Acetyl-1,2:5,6-di-O-isopropylidene-3-thio.alpha.d-allofuranose	0.03
194	37.04	Dodecanoic acid, 3,7,11-trimethyl-, methyl ester	0.02
195	37.13	Adipic acid, 4-methoxy-2-methylbutyl tetradecyl ester	0.06
196	37.28	Benzenepropanoic acid, 4-benzyloxy-, ethyl ester	0.05
198	37.53	Z-14-Octadecen-1-ol acetate	0.03
200	37.74	Bis(2-ethylhexyl) phthalate	0.5
202	37.97	1-(4-Amino-furazan-3-yl)-5-[(benzyl-methyl-amino)-methyl]-1H-[1,2,3]triazole-4-carboxylic acid ethyl ester	0.22
205	38.39	9H-Benzo[4,5]imidazo[2,1-c][1,2,4]triazole, 3-benzylsulfanyl-	0.79
207	38.63	Cyclohexane, 1-ethenyl-3-methylene-5-(1-propenylidene)-	0.01
208	38.72	2,6-Lutidine-4-[benzylamino]-3,5-dichloro-	0.07
210	38.88	1-Methoxy-4-dimethyl(trimethylsilylmethyl)silyloxymethylbenzene	0.14
212	39.45	13-Docosenamide, (Z)-	0.15
214	39.7	Benzonitrile, 3-fluoro-	0.03
227	42.02	Dihydrotachysterol	0.11
235	44.11	4-Methoxybenzylamine, N,N-dinonyl-	0.02
236	44.58	.gamma.-Sitosterol	0.08
242	48.73	3-(2-Ethyl-piperidin-1-ylmethyl)-8a-methyl-5-methylene-decahydro-naphtho[2,3-b]furan-2-one	0.11
