# Supplementary material for: Research on using Aquilaria sinensis callus to evaluate the agarwood-inducing potential of fungi
Source: PLoS One. 2024 Dec 26;19(12):e0316178. doi: 10.1371/journal.pone.0316178 (PMC11671001; doi:10.1371/journal.pone.0316178)
Supplement: Attachment — Submitted filename: Rebuttal letter.docx [file pone.0316178.s005.docx]

Dear editor,

Thank you very much for your kind letter and the reviewers’ comments concerning our manuscript entitled 'Screening of agarwood-inducing fungi by the callus of *Aquilaria sinensis*' (ID: PONE-D-24-13425). The comments were very valuable and helpful for revising and improving our paper as well as for guiding our research. We have thoroughly considered the comments and substantially revised our manuscript. We have highlighted the revisions in the manuscript in red.

**The point-by-point replies to the editor's comments are listed below.**

**Editor's Comments & Our Responses:**

**1.** Please ensure that your manuscript meets PLOS ONE's style requirements, including those for file naming.

**Response:** Thank you for the comment. We have carefully reviewed the manuscript format and ensured that it meets the required standards. Thanks again for your helpful suggestions!

**2.** In your Methods section, please provide additional information regarding the permits you obtained for the work. Please ensure you have included the full name of the authority that approved the field site access and, if no permits were required, a brief statement explaining why.

**Response:** Thank you for the comment. The *Aquilaria sinensis* trees involved in this study were cultivated in our laboratory, so no additional field permits were required. Thanks again for your helpful suggestions!

**3.** Thank you for stating the following financial disclosure: “This research was funded by National Key R & D Program of China (2023YFD1401304).” Please state what role the funders took in the study. If the funders had no role, please state: "The funders had no role in study design, data collection and analysis, decision to publish, or preparation of the manuscript." If this statement is not correct you must amend it as needed. Please include this amended Role of Funder statement in your cover letter; we will change the online submission form on your behalf.

**Response:** Thank you for the comment. The funders provided financial support for the experiment but did not directly participate in the research. Therefore, we declare that 'The funders had no role in study design, data collection and analysis, decision to publish, or preparation of the manuscript.' Thanks again for your helpful suggestions!

**4.** Thank you for stating the following in the Acknowledgments Section of your manuscript: “This research was funded by National Key R & D Program of China (2023YFD1401304).” We note that you have provided funding information that is currently declared in your Funding Statement. However, funding information should not appear in the Acknowledgments section or other areas of your manuscript. We will only publish funding information present in the Funding Statement section of the online submission form. Please remove any funding-related text from the manuscript and let us know how you would like to update your Funding Statement. Currently, your Funding Statement reads as follows: “This research was funded by National Key R & D Program of China (2023YFD1401304).” Please include your amended statements within your cover letter; we will change the online submission form on your behalf.

**Response:** Thank you for the comment. We have removed the relevant content from the manuscript. Thanks again for your helpful suggestions!

**5.** We note that your Data Availability Statement is currently as follows: [All relevant data are within the manuscript and its Supporting Information files. Please confirm at this time whether or not your submission contains all raw data required to replicate the results of your study. Authors must share the “minimal data set” for their submission. PLOS defines the minimal data set to consist of the data required to replicate all study findings reported in the article, as well as related metadata and methods.

**Response:** Thank you for the comment. We have uploaded the raw data, and here is the link: <https://doi.org/10.6084/m9.figshare.26788321.v1> Thanks again for your helpful suggestions!

**The point-by-point replies to the reviewers’ comments are listed below.**

**Reviewers' Comments & Our Responses:**

**Reviewer #1:**

*Aquilaria sinensis* can produced agarwood induced by fungi, wound and so on. To find which species of fungi can high effective induce agarwood formation is important. In this manuscript, the authors revealed two fungi can changed the superoxide dismutase (SOD) and peroxidase (POD) activities as well as gene expression related to secondary metabolites using Quantitative Real-time PCR. However, there are some points need to be further considered.

**Response:** Special thanks to the reviewer for the positive evaluation.

**1.**The title can’t match the studies carried out in this manuscript.

**Response:** Thank you for the comment. We changed the title as：“ Research on Using *Aquilaria sinensis* Callus to Evaluate the Agarwood-Inducing Potential of Fungi ”Thanks again for your helpful suggestions!

**2.**The writing is very confused. In abstract, the experiments were not clearly described such as what is W-1 and W-15 (line 21). The writing of genes is wrong.

**Response:** Thank you for the comment. We acknowledge that the expression in the abstract may not have been clear, especially regarding the descriptions of W-1 and W-15. There were also errors in the gene nomenclature, for which we sincerely apologize. Therefore, we have rewritten the abstract to ensure that the experimental design and key points are clearly articulated. The gene names will be written in the correct standard format.

The revised abstract is as follows: “Agarwood is a precious resinous heartwood highly valued for its cultural, religious, and medicinal significance. With the increasing market demand, natural agarwood resources are rapidly depleting, making the development of effective artificial induction methods for agarwood highly significant. This study aims to explore the feasibility of using callus tissue to assess the ability of fungi to induce agarwood formation. We selected two fungi isolated from *Aquilaria sinensis*, W-1 (*Podospora setosa*) and W-15 (*Alternaria alstroemeriae*), and used the known agarwood-inducing fungi YMY (*Pestalotiopsis* sp.) as a positive control, by treating *A. sinensis* callus with their fermented filtrates. The experimental results showed that W-1 and W-15 treatments significantly enhanced the activity of superoxide dismutase (SOD) and peroxidase (POD) in the callus tissue and upregulated the expression of 3-hydroxy-3-methylglutaryl-CoA synthase (HMGS), 1-deoxy-D-xylulose-5-phosphate reductoisomerase (DXR), and sesquiterpene synthase (ASS-1). The experimental results showed that W-1 and W-15 treatments significantly enhanced the activity of superoxide dismutase (SOD) and peroxidase (POD) in the callus tissue and upregulated the expression of 3-hydroxy-3-methylglutaryl-CoA synthase (HMGS), 1-deoxy-D-xylulose-5-phosphate reductoisomerase (DXR), and sesquiterpene synthase (ASS-1). In conclusion, this study demonstrates that using callus tissue to screen fungi capable of inducing agarwood is feasible and effective, providing new insights for optimizing agarwood production techniques in the future.”

**3.**The introduction is too long and missed the main points. It needs to be focused on how fungi introduce the agarwood formation and why you carried out superoxide dismutase (SOD) and peroxidase (POD) activities. Why selected these genes to carried out by Quantitative Real-time PCR.

**Response:** Thank you for the comment. We have revised the introduction and provided an explanation for why we conducted the superoxide dismutase (SOD) and peroxidase (POD) activity studies, as well as the reasons for selecting these genes for Quantitative Real-time PCR analysis.

“Fungi, as the key factor in inducing agarwood formation, stimulate agarwood trees to produce defensive secondary metabolites through intricate physiological and biochemical mechanisms. In a natural environment, when agarwood trees are infected by fungi, the tree recognizes the fungi invasion and activates its defense response, a process considered the starting point of agarwood formation [9]. The core of the defense response lies in inducing stress responses through signaling pathways, leading to enhanced antioxidant enzyme activity, triggering the expression of a series of disease-resistant genes, and ultimately resulting in the accumulation of secondary metabolites [2]. These metabolites, including sesquiterpenes and 2-(2-phenylethyl) chromones, are the main active components of agarwood [10].

Fungi infection is often accompanied by oxidative stress responses in plant cells. Plant cells counter pathogen invasion by producing reactive oxygen species (ROS) such as superoxide anions (O₂⁻) [11]. To mitigate the toxicity of ROS, the antioxidant enzyme system within agarwood trees is rapidly activated. Superoxide dismutase (SOD), peroxidase (POD), and catalase (CAT) are the main antioxidant enzymes involved, working together to eliminate excess ROS and maintain redox balance within cells [12]. The enhancement of these enzyme activities is not only a direct response of plants to fungi invasion but also indirectly promotes the activation of secondary metabolic pathways related to agarwood formation [9]. Under fungi induction, the secondary metabolic pathways within agarwood trees are significantly activated, particularly the mevalonate (MVA) pathway and the methylerythritol phosphate (MEP) pathway. 3-Hydroxy-3-methylglutaryl-CoA synthase (HMGS) and 1-deoxy-D-xylulose-5-phosphate reductoisomerase (DXR) are the key rate-limiting enzymes in the MVA and MEP pathways, respectively [13, 14]. The upregulated expression of these enzymes promotes the increased synthesis of sesquiterpene compounds. ASS-1 is a typical wound-induced gene responsible for sesquiterpene formation in agarwood. Studies have found that ASS-1 expression is barely detectable in healthy *A. sinensis* callus or cell cultures, but its expression significantly increases in wounded tissues or jasmonic acid methyl ester-treated callus, leading to a corresponding increase in sesquiterpene compounds [15]. Fungi infection can significantly increase the accumulation of secondary metabolites in agarwood trees by regulating the gene expression of defense enzymes and these key enzymes, thereby enhancing the yield and quality of agarwood.”

**4.**The study isolated fungi from A. sinensis in Chengmai County, Hainan Province, China. How and why to isolated them from Chengmai County?

**Response:** Thank you for the comment. The reason for isolating fungi from Chengmai County is that Chengmai is more suitable for the growth of *Aquilaria sinensis* compared to Changsha. Therefore, the fungi were isolated from Chengmai County. The method used for isolating the fungi was:

“After culturing on Potato Dextrose Agar (PDA) plates at 25°C for 7 days, single fungi isolates were purified using hyphal tip isolation technique.”

**5.**These fungi were isolated and preserved by the Key Laboratory of National Forestry and Grassland Administration on Control of Artiffcial Forest Diseases and Pests in South China (Changsha, China). What is the difference between the fungi from Chengmai County? Why did it differently?

**Response:** Thank you for the comment. There is no difference. Due to the lack of experimental conditions in Chengmai County, the fungi were isolated and preserved at the Key Laboratory of Forest Pest Control for Southern Plantations, National Forestry and Grassland Administration, in Changsha, China.

**6.**Why set the experiments as W-1, W-15, and YMY (CK2), as well as sterile PDA (CK). What is the YMY (CK2), as well as sterile PDA (CK) and why?

**Response:** Thank you for the comment. W-1 and W-15 were used as experimental groups because their ability to induce agarwood formation was unknown. YMY, a fungi known for its ability to promote agarwood formation, served as the positive control, and sterile PDA was used to exclude any effects of the culture medium on the experiment. A more detailed description has also been provided in the manuscript.

“In this study, we selected the fungi W-1 and W-15, whose ability to promote agarwood formation was not previously established, and used the known agarwood-inducing fungi YMY [19] as a positive control.”

**7.**What is the logic relationship between the callus of A. sinensis and the trees? Are the results of callus suitable for the tree?

**Response:** Thank you for the comment. The logical relationship between the *Aquilaria sinensis* callus and the tree is described in the abstract.

“The callus of *A. sinensis* is an undifferentiated cell mass induced from plant tissue through in vitro culture, and it shares physiological and biochemical similarities with the whole tree. The metabolic pathways and stress responses of callus tissue largely retain the characteristics of the tree, making it a common model for studying the formation mechanisms of agarwood [16, 17]. However, because callus tissue grows in a controlled environment, lacking the complex structure and environmental interactions found in mature trees, its research findings may require further validation and optimization when applied to actual trees [18].”

**8.**The repeats of the experiments are unclear.

**Response:** Thank you for the comment. The number of replicates for each experiment, which was three, is detailed in the methods section of the manuscript.

**9.**The results and conclusion need to be reorganized and make it sense.

**Response:** Thank you for the comment. We have reorganized the results and conclusions and also made revisions to the discussion section.

**Effect of two fungi on SOD activity in callus of *A. sinensis***

“In this study, the SOD activity in callus treated with W-1 and W-15 showed an initial increase followed by a decrease, with the SOD activity in W-1 treatment peaking at 457.36 U/gmin^-1^ at 48 h, and W-15 treatment peaking at 474.49 U/gmin^-1^ at 8 h. This pattern of change is consistent with previous studies; in contrast, the SOD activity in YMY-treated callus initially decreased, but significantly increased after 24 h, reaching a peak of 388.89 U/gmin^-1^ (Fig 3). This differing pattern of change may be related to the different active components or strains in the YMY fermentation filtrate. It is worth noting that the SOD activity in the sterilized PDA treatment showed no significant change, further confirming that W-1, W-15, and YMY all can induce changes in SOD activity in *A. sinensis* callus.”

**Effect of two fungi on POD activity in callus of *A. sinensis***

“In this study, the POD activity in callus treated with W-1 peaked at 220.50 U/gmin^-1^ at 12 h. In contrast, the POD activity in W-15 treated callus reached a maximum of 211.16 U/gmin^-1^ at 48 h. Differently, the POD activity in YMY-treated callus peaked at 4 h, then decreased, but rose again to the highest value of 230.83 U/gmin^-1^ at 48 h (Fig 4). The results indicate that both fungi could significantly alter POD activity in *A. sinensis* callus.”

**Verification of the ability of two fungi to induce agarwood formation.**

“To further verify whether *Aquilaria sinensis* callus can be used to screen for agarwood-inducing fungi, the samples were treated with W-1, W-15, and YMY (CK) fermentation filtrates for six months, resulting in a color change (Fig 4). GC-MS analysis revealed that 231, 305, and 242 compounds were detected in the samples treated with W-1, W-15, and YMY (CK), respectively (Supplementary Tables S1, S2, S3). The content of sesquiterpenes in the essential oils treated with W-1 and W-15 was 9.76% and 16.69%, respectively, while the content in the control group was 10.12% (Table 2). Four terpenoid compounds were common to all samples: Dehydrofukinone, Alloaromadendrene, Cycloheptane, 1-ethenyl-1-methyl-4-methylene-2-(2-methyl-1-propenyl)-, and Squalene.。Among them, Dehydrofukinone and Alloaromadendrene exhibit antioxidant and antimicrobial activities [23-26], while Squalene is a triterpene with various applications in the food, chemical, and healthcare industries [27]. Aromatic compounds also contribute to the distinctive fragrance of agarwood. The content of aromatic compounds in the essential oils treated with W-1 and W-15 was 21.61% and 21.6%, respectively, compared to 16.66% in the control group. Benzylacetone, which has antitussive, expectorant, and antiasthmatic effects [28], was detected in both the treatment and control groups. The content of chromone compounds was relatively low in the essential oils treated with W-1 and W-15, at 3.07% and 3.17%, respectively, compared to 4.38% in the control group. The experimental results indicate that W-1 and W-15 have the ability to induce agarwood formation.”

**Conclusions**

“This study explored the induction effects of W-1 and W-15 on agarwood formation by inoculating *Aquilaria sinensis* callus tissues with different fungi fermentation broths. The experimental results showed that W-1 and W-15 treatments significantly enhanced the activity of superoxide dismutase (SOD) and peroxidase (POD) in the callus tissues, while also upregulating the expression of key metabolic enzymes such as HMGS, DXR, and ASS-1. These changes suggest that W-1 and W-15 can promote the accumulation of agarwood-related compounds by activating antioxidant defense mechanisms and secondary metabolic pathways. Moreover, GC-MS analysis revealed a significant increase in the content of sesquiterpenes and aromatic compounds in the samples treated with W-1 and W-15, further validating the effectiveness of these two fungi in inducing agarwood formation. Therefore, this study demonstrates that using callus tissue as a method for rapidly screening fungi that promote agarwood formation is feasible and effective. This method not only offers new insights”

10.The figures need to be reorganized to be more informative. Such as, only one figure in figure 1, why set it? What did the authors mean?

**Response:** Thank you for the comment. Figure 1 shows the phylogenetic tree of W-1, and Figure 2 shows the phylogenetic tree of W-15. The titles will be highlighted when uploading the images. Thanks again for your helpful suggestions!

Finally, we would like to thank you once again for your valuable comments.

**Reviewer #2:**

**1.** Lacks Novelty

**Response:** Thank you for the comment. We understand the reviewers' concern about the novelty of the study. During the revision process, we will emphasize the innovative aspects of our research, particularly the new insights reflected in the experimental design and result analysis. The introduction and discussion sections will be revised to highlight our study's unique contributions, including how examining SOD and POD activities and secondary metabolic gene expression reveals the potential mechanisms by which fungi influence agarwood formation.

**2.** Need some more experimentation

**Response:** Thank you for the comment. We understand the reviewers' suggestion to add more experiments. However, we regret that due to the limitation of experimental materials, it is not easy to add additional experiments. Nevertheless, we have conducted a more thorough analysis of the existing experiments and have improved the introduction and discussion sections, hoping to meet the standards for publication.

**3.** The manuscript doesn't highlight what new knowledge has been acquired with the set of experiments performed

**Response:** Thank you for the comment. In the revised manuscript, we will more clearly explain the new findings obtained through these experiments and their contribution to the existing literature. We have improved the abstract, introduction, discussion, and results sections to highlight the significance of this study. The following are the revisions made:

**Abstract:**

“Agarwood is a precious resinous heartwood highly valued for its cultural, religious, and medicinal significance. With the increasing market demand, natural agarwood resources are rapidly depleting, making the development of effective artificial induction methods for agarwood highly significant. This study aims to explore the feasibility of using callus tissue to assess the ability of fungi to induce agarwood formation. We selected two fungi isolated from *Aquilaria sinensis*, W-1 (*Podospora setosa*) and W-15 (*Alternaria alstroemeriae*), and used the known agarwood-inducing fungi YMY (*Pestalotiopsis* sp.) as a positive control, by treating *Aquilaria sinensis* callus with their fermented filtrates. The experimental results showed that W-1 and W-15 treatments significantly enhanced the activity of superoxide dismutase (SOD) and peroxidase (POD) in the callus tissue and upregulated the expression of 3-hydroxy-3-methylglutaryl-CoA synthase (HMGS), 1-deoxy-D-xylulose-5-phosphate reductoisomerase (DXR), and sesquiterpene synthase (ASS-1). The experimental results showed that W-1 and W-15 treatments significantly enhanced the activity of superoxide dismutase (SOD) and peroxidase (POD) in the callus tissue and upregulated the expression of 3-hydroxy-3-methylglutaryl-CoA synthase (HMGS), 1-deoxy-D-xylulose-5-phosphate reductoisomerase (DXR), and sesquiterpene synthase (ASS-1). In conclusion, this study demonstrates that using callus tissue to screen fungi capable of inducing agarwood is feasible and effective, providing new insights for optimizing agarwood production techniques in the future.”

**Introduction**

“Fungi, as the key factor in inducing agarwood formation, stimulate agarwood trees to produce defensive secondary metabolites through intricate physiological and biochemical mechanisms. In a natural environment, when agarwood trees are infected by fungi, the tree recognizes the fungi invasion and activates its defense response, a process considered the starting point of agarwood formation [9]. The core of the defense response lies in inducing stress responses through signaling pathways, leading to enhanced antioxidant enzyme activity, triggering the expression of a series of disease-resistant genes, and ultimately resulting in the accumulation of secondary metabolites [2]. These metabolites, including sesquiterpenes and 2-(2-phenylethyl) chromones, are the main active components of agarwood [10].

Fungi infection is often accompanied by oxidative stress responses in plant cells. Plant cells counter pathogen invasion by producing reactive oxygen species (ROS) such as superoxide anions (O₂⁻) [11]. To mitigate the toxicity of ROS, the antioxidant enzyme system within agarwood trees is rapidly activated. Superoxide dismutase (SOD), peroxidase (POD), and catalase (CAT) are the main antioxidant enzymes involved, working together to eliminate excess ROS and maintain redox balance within cells [12]. The enhancement of these enzyme activities is not only a direct response of plants to fungi invasion but also indirectly promotes the activation of secondary metabolic pathways related to agarwood formation [9]. Under fungi induction, the secondary metabolic pathways within agarwood trees are significantly activated, particularly the mevalonate (MVA) pathway and the methylerythritol phosphate (MEP) pathway. 3-Hydroxy-3-methylglutaryl-CoA synthase (HMGS) and 1-deoxy-D-xylulose-5-phosphate reductoisomerase (DXR) are the key rate-limiting enzymes in the MVA and MEP pathways, respectively [13, 14]. The upregulated expression of these enzymes promotes the increased synthesis of sesquiterpene compounds. ASS-1 is a typical wound-induced gene responsible for sesquiterpene formation in agarwood. Studies have found that ASS-1 expression is barely detectable in healthy *A. sinensis* callus or cell cultures, but its expression significantly increases in wounded tissues or jasmonic acid methyl ester-treated callus, leading to a corresponding increase in sesquiterpene compounds [15]. Fungi infection can significantly increase the accumulation of secondary metabolites in agarwood trees by regulating the gene expression of defense enzymes and these key enzymes, thereby enhancing the yield and quality of agarwood.

The callus of *A. sinensis* is an undifferentiated cell mass induced from plant tissue through in vitro culture, and it shares physiological and biochemical similarities with the whole tree. The metabolic pathways and stress responses of callus tissue largely retain the characteristics of the tree, making it a common model for studying the formation mechanisms of agarwood [16, 17]. However, because callus tissue grows in a controlled environment, lacking the complex structure and environmental interactions found in mature trees, its research findings may require further validation and optimization when applied to actual trees [18]. Therefore, we consider whether callus tissue can be used to screen fungi that promote agarwood formation.

In this study, we selected the fungi W-1 and W-15, whose ability to promote agarwood formation was not previously established, and used the known agarwood-inducing fungi YMY [19] as a positive control. We treated the callus tissue and preliminarily explored the feasibility of using callus tissue to assess the ability of fungi to induce agarwood formation, providing a new approach for screening fungi that induce agarwood.”

**Discussion**

“Due to the slow and unpredictable process of agarwood formation in nature, recent research has increasingly focused on the artificial production of agarwood through fungi induction. According to the latest research statistics, only 59 strains from 16 different genera of fungi have been reported to have the ability to induce agarwood formation [29]. Therefore, identifying fungi species capable of efficiently inducing agarwood formation is of great significance.

It has been confirmed that fungi can regulate the production of metabolites by affecting the activity of plant defense enzymes. Zhu et al. used the endophytic fungi GG22 (*Fusarium oxysporum*) to treat *Rehmannia*, which increased the SOD and POD activities in *Rehmannia*, activated or enhanced the expression of related enzymes in primary metabolic pathways, and thereby increased the content of secondary metabolites [30]. Changes in defense enzyme activity are often accompanied by the accumulation of specific secondary metabolites. In the process of agarwood formation, fungi infection induces the activity of antioxidant and defense enzymes, promoting the synthesis of terpenoids and phenylpropanoids, which are key aromatic compounds in agarwood. Similarly, in other plants, fungi infection can promote the accumulation of metabolites such as flavonoids, coumarins, and alkaloids by regulating the activity of defense enzymes [12, 31]. In this study, the SOD and POD activities in callus tissues treated with W-1 and W-15 showed an initial increase followed by a decline, consistent with previous studies, suggesting that W-1 and W-15 may promote agarwood formation by stimulating the antioxidant defense mechanisms in the callus tissues [5].

Numerous studies have shown that fungi can enhance the production of secondary metabolites by regulating the expression of key structural genes and regulatory genes involved in their biosynthesis. Liu et al. found that the fungi *Phaeoacremonium rubrigenum* increases sesquiterpene biosynthesis by upregulating enzymes in the MVA pathway of *Aquilaria sinensis* [32]. Zhang et al. showed that methyl jasmonate and hydrogen peroxide can increase the expression of the *Sa*DXR gene in *Santalum album*, thereby enhancing the biosynthesis of sesquiterpenes [33]. In this study, treatment with W-1 and W-15 significantly increased the expression levels of the HMGS and DXR genes in the callus tissues of *Aquilaria sinensis*. This upregulation might be part of the callus's defense response to fungi infection, thereby promoting agarwood formation [32]. The expression pattern of the ASS-1 gene was consistent with previous findings, showing minimal expression in callus tissues treated with sterile PDA but significant expression following W-1 and W-15 treatments. The experimental results showed that the activities of POD, SOD, and the expression levels of the ASS-1, HMGS, and DXR genes in the experimental groups and the positive control group were all higher than those in the negative control group, indicating that W-1 and W-15 have similar capabilities to YMY in inducing agarwood production.

The effectiveness of these fungi in inducing agarwood formation was further confirmed by GC-MS analysis. The results showed a significant increase in the variety of volatile oil compounds in *Aquilaria sinensis* treated with W-1 and W-15, particularly in the content of sesquiterpenes and aromatic compounds. The sesquiterpene content in the W-1 treated samples was 9.76%, in W-15 treated samples 16.69%, compared to 10.12% in the control group. These results suggest that W-1 and W-15 can effectively induce agarwood formation and enhance its quality by activating the metabolic pathways of *Aquilaria sinensis* [34]. Moreover, the content of aromatic compounds in the W-1 and W-15 treated samples was significantly higher than in the control group, further confirming the effectiveness of these fungi in promoting agarwood formation. The results confirmed that fungi W-1 and W-15, similar to YMY, possess the ability to induce agarwood production. In conclusion, using callus tissue to assess the ability of fungi to induce agarwood formation is a rapid and reliable method. This approach not only aids in enriching fungi resources that promote agarwood formation but also helps protect wild agarwood.”

**Conclusions**

“This study explored the induction effects of W-1 and W-15 on agarwood formation by inoculating *Aquilaria sinensis* callus tissues with different fungi fermentation broths. he experimental results showed that W-1 and W-15 treatments significantly enhanced the activity of superoxide dismutase (SOD) and peroxidase (POD) in the callus tissues, while also upregulating the expression of key metabolic enzymes such as HMGS, DXR, and ASS-1. These changes suggest that W-1 and W-15 can promote the accumulation of agarwood-related compounds by activating antioxidant defense mechanisms and secondary metabolic pathways. Moreover, GC-MS analysis revealed a significant increase in the content of sesquiterpenes and aromatic compounds in the samples treated with W-1 and W-15, further validating the effectiveness of these two fungi in inducing agarwood formation. Therefore, this study demonstrates that using callus tissue as a method for rapidly screening fungi that promote agarwood formation is feasible and effective. This method not only offers new insights for screening fungi resources that efficiently induce agarwood formation but also contributes to the protection and sustainable use of wild agarwood resources.”

**4.** The fungi used in the study are not from the Plant-Microbe interaction microbiota, so explanation needed for using them at length, which is totally missing.

**Response:** Thank you for the comment. We acknowledge the reviewers' concerns regarding the use of fungi that are not from the plant-microbe interaction microbiota. In the revised manuscript, we will provide a detailed explanation of the rationale for selecting these fungi, their relationship with *Aquilaria sinensis*, and the significance of the experiments. Additionally, we will supplement the methods and discussion sections with a thorough explanation of the fungi's origin and their applicability in agarwood formation studies, clarifying why these fungi, despite not being part of the typical plant-microbe interaction microbiota, still hold research value.

**5.** Only one core sesquiterpenoid gene expression has been studied, why?

**Response:** Thank you for the comment. The reason for choosing ASS-1 is because “ASS-1 is a typical wound-induced gene responsible for sesquiterpene formation in agarwood. Studies have found that ASS-1 expression is barely detectable in healthy *A. sinensis* callus or cell cultures, but its expression significantly increases in wounded tissues or jasmonic acid methyl ester-treated callus, leading to a corresponding increase in sesquiterpene compounds [15].”

In addition to ASS-1, we also selected the key rate-limiting enzymes HNGS and DXR from the MVA pathway and the MEP pathway for our study

**6.**The study is very basic in nature and needs some more value addition to meet the standards of the journal.

**Response:** Thank you for the comment. We have strived to deepen the study through more detailed analysis and to enhance the scientific value of the manuscript to meet the journal's standards. During the revision process, we refined the abstract and discussion to increase the complexity and scientific contribution of the research, ensuring that it not only has a foundational basis but also makes a practical advancement in the field.

Finally, we would like to thank you once again for your valuable comments.

**Reviewer #3:**

The data is not well correlated and statistically analyze. Discussion too surface and not well justified. Correlation of the fungi with chemical data is required for more scientifically discuss. Need more improvement in preparing the manuscript to be cited.

**Response:** We appreciate the reviewers' thorough review of our manuscript and their constructive feedback. We highly value these suggestions and have made corresponding adjustments during the revision process. Below is our response to the specific comments provided by the reviewers:

**1.Data Correlation and Statistical Analysis**

We recognize the importance of data correlation and statistical analysis. To address this, we have re-examined all the data and conducted more detailed statistical analyses. In the results and discussion sections, we have supplemented the manuscript with these detailed statistical analyses and provided thorough explanations of the findings, thereby enhancing the scientific rigor and robustness of the study.

2. **Depth of Discussion and Argumentation**

We agree that the discussion section requires deeper analysis and stronger argumentation. To address this, we have expanded the discussion section, providing a more in-depth analysis of the results, including comparisons with existing literature. Additionally, we further elaborated on the scientific significance of our findings and their contribution to understanding the mechanisms of agarwood formation.

**3. Correlation Between Fungi and Chemical Data** We understand the reviewer's suggestion to better correlate the fungi with the chemical data. We have added a discussion on how the fungi influence the changes in secondary metabolites of *Aquilaria sinensis*.

**4.Overall Improvement of the Manuscript** **Response**: We recognize that there is room for improvement in the expression and organization of the manuscript. Therefore, we have rewritten the manuscript to ensure it meets the standards required for publication.

Finally, we would like to thank you once again for your valuable comments.

The following are the revisions made to the manuscript:

**Abstract:**

“Agarwood is a precious resinous heartwood highly valued for its cultural, religious, and medicinal significance. With the increasing market demand, natural agarwood resources are rapidly depleting, making the development of effective artificial induction methods for agarwood highly significant. This study aims to explore the feasibility of using callus tissue to assess the ability of fungi to induce agarwood formation. We selected two fungi isolated from *Aquilaria sinensis*, W-1 (*Podospora setosa*) and W-15 (*Alternaria alstroemeriae*), and used the known agarwood-inducing fungi YMY (*Pestalotiopsis* sp.) as a positive control, by treating *Aquilaria sinensis* callus with their fermented filtrates. The experimental results showed that W-1 and W-15 treatments significantly enhanced the activity of superoxide dismutase (SOD) and peroxidase (POD) in the callus tissue and upregulated the expression of 3-hydroxy-3-methylglutaryl-CoA synthase (HMGS), 1-deoxy-D-xylulose-5-phosphate reductoisomerase (DXR), and sesquiterpene synthase (ASS-1). The experimental results showed that W-1 and W-15 treatments significantly enhanced the activity of superoxide dismutase (SOD) and peroxidase (POD) in the callus tissue and upregulated the expression of 3-hydroxy-3-methylglutaryl-CoA synthase (HMGS), 1-deoxy-D-xylulose-5-phosphate reductoisomerase (DXR), and sesquiterpene synthase (ASS-1). In conclusion, this study demonstrates that using callus tissue to screen fungi capable of inducing agarwood is feasible and effective, providing new insights for optimizing agarwood production techniques in the future.”

**Introduction**

“Fungi, as the key factor in inducing agarwood formation, stimulate agarwood trees to produce defensive secondary metabolites through intricate physiological and biochemical mechanisms. In a natural environment, when agarwood trees are infected by fungi, the tree recognizes the fungi invasion and activates its defense response, a process considered the starting point of agarwood formation [9]. The core of the defense response lies in inducing stress responses through signaling pathways, leading to enhanced antioxidant enzyme activity, triggering the expression of a series of disease-resistant genes, and ultimately resulting in the accumulation of secondary metabolites [2]. These metabolites, including sesquiterpenes and 2-(2-phenylethyl) chromones, are the main active components of agarwood [10].

Fungi infection is often accompanied by oxidative stress responses in plant cells. Plant cells counter pathogen invasion by producing reactive oxygen species (ROS) such as superoxide anions (O₂⁻) [11]. To mitigate the toxicity of ROS, the antioxidant enzyme system within agarwood trees is rapidly activated. Superoxide dismutase (SOD), peroxidase (POD), and catalase (CAT) are the main antioxidant enzymes involved, working together to eliminate excess ROS and maintain redox balance within cells [12]. The enhancement of these enzyme activities is not only a direct response of plants to fungi invasion but also indirectly promotes the activation of secondary metabolic pathways related to agarwood formation [9]. Under fungi induction, the secondary metabolic pathways within agarwood trees are significantly activated, particularly the mevalonate (MVA) pathway and the methylerythritol phosphate (MEP) pathway. 3-Hydroxy-3-methylglutaryl-CoA synthase (HMGS) and 1-deoxy-D-xylulose-5-phosphate reductoisomerase (DXR) are the key rate-limiting enzymes in the MVA and MEP pathways, respectively [13, 14]. The upregulated expression of these enzymes promotes the increased synthesis of sesquiterpene compounds. ASS-1 is a typical wound-induced gene responsible for sesquiterpene formation in agarwood. Studies have found that ASS-1 expression is barely detectable in healthy *A. sinensis* callus or cell cultures, but its expression significantly increases in wounded tissues or jasmonic acid methyl ester-treated callus, leading to a corresponding increase in sesquiterpene compounds [15]. Fungi infection can significantly increase the accumulation of secondary metabolites in agarwood trees by regulating the gene expression of defense enzymes and these key enzymes, thereby enhancing the yield and quality of agarwood.

The callus of *Aquilaria sinensis* is an undifferentiated cell mass induced from plant tissue through in vitro culture, and it shares physiological and biochemical similarities with the whole tree. The metabolic pathways and stress responses of callus tissue largely retain the characteristics of the tree, making it a common model for studying the formation mechanisms of agarwood [16, 17]. However, because callus tissue grows in a controlled environment, lacking the complex structure and environmental interactions found in mature trees, its research findings may require further validation and optimization when applied to actual trees [18]. Therefore, we consider whether callus tissue can be used to screen fungi that promote agarwood formation.

In this study, we selected the fungi W-1 and W-15, whose ability to promote agarwood formation was not previously established, and used the known agarwood-inducing fungi YMY [19] as a positive control. We treated the callus tissue and preliminarily explored the feasibility of using callus tissue to assess the ability of fungi to induce agarwood formation, providing a new approach for screening fungi that induce agarwood.”

**Discussion**

“Due to the slow and unpredictable process of agarwood formation in nature, recent research has increasingly focused on the artificial production of agarwood through fungi induction. According to the latest research statistics, only 59 strains from 16 different genera of fungi have been reported to have the ability to induce agarwood formation [29]. Therefore, identifying fungi species capable of efficiently inducing agarwood formation is of great significance.

It has been confirmed that fungi can regulate the production of metabolites by affecting the activity of plant defense enzymes. Zhu et al. used the endophytic fungi GG22 (*Fusarium oxysporum*) to treat *Rehmannia*, which increased the SOD and POD activities in *Rehmannia*, activated or enhanced the expression of related enzymes in primary metabolic pathways, and thereby increased the content of secondary metabolites [30]. Changes in defense enzyme activity are often accompanied by the accumulation of specific secondary metabolites. In the process of agarwood formation, fungi infection induces the activity of antioxidant and defense enzymes, promoting the synthesis of terpenoids and phenylpropanoids, which are key aromatic compounds in agarwood. Similarly, in other plants, fungi infection can promote the accumulation of metabolites such as flavonoids, coumarins, and alkaloids by regulating the activity of defense enzymes [12, 31]. In this study, the SOD and POD activities in callus tissues treated with W-1 and W-15 showed an initial increase followed by a decline, consistent with previous studies, suggesting that W-1 and W-15 may promote agarwood formation by stimulating the antioxidant defense mechanisms in the callus tissues [5].

Numerous studies have shown that fungi can enhance the production of secondary metabolites by regulating the expression of key structural genes and regulatory genes involved in their biosynthesis. Liu et al. found that the fungi *Phaeoacremonium rubrigenum* increases sesquiterpene biosynthesis by upregulating enzymes in the MVA pathway of *Aquilaria sinensis* [32]. Zhang et al. showed that methyl jasmonate and hydrogen peroxide can increase the expression of the *Sa*DXR gene in *Santalum album*, thereby enhancing the biosynthesis of sesquiterpenes [33]. In this study, treatment with W-1 and W-15 significantly increased the expression levels of the HMGS and DXR genes in the callus tissues of *Aquilaria sinensis*. This upregulation might be part of the callus's defense response to fungi infection, thereby promoting agarwood formation [32]. The expression pattern of the ASS-1 gene was consistent with previous findings, showing minimal expression in callus tissues treated with sterile PDA but significant expression following W-1 and W-15 treatments. The experimental results showed that the activities of POD, SOD, and the expression levels of the ASS-1, HMGS, and DXR genes in the experimental groups and the positive control group were all higher than those in the negative control group, indicating that W-1 and W-15 have similar capabilities to YMY in inducing agarwood production.

The effectiveness of these fungi in inducing agarwood formation was further confirmed by GC-MS analysis. The results showed a significant increase in the variety of volatile oil compounds in *Aquilaria sinensis* treated with W-1 and W-15, particularly in the content of sesquiterpenes and aromatic compounds. The sesquiterpene content in the W-1 treated samples was 9.76%, in W-15 treated samples 16.69%, compared to 10.12% in the control group. These results suggest that W-1 and W-15 can effectively induce agarwood formation and enhance its quality by activating the metabolic pathways of *Aquilaria sinensis* [34]. Moreover, the content of aromatic compounds in the W-1 and W-15 treated samples was significantly higher than in the control group, further confirming the effectiveness of these fungi in promoting agarwood formation. The results confirmed that fungi W-1 and W-15, similar to YMY, possess the ability to induce agarwood production. In conclusion, using callus tissue to assess the ability of fungi to induce agarwood formation is a rapid and reliable method. This approach not only aids in enriching fungi resources that promote agarwood formation but also helps protect wild agarwood.”

**Conclusions**

“This study explored the induction effects of W-1 and W-15 on agarwood formation by inoculating *Aquilaria sinensis* callus tissues with different fungi fermentation broths. he experimental results showed that W-1 and W-15 treatments significantly enhanced the activity of superoxide dismutase (SOD) and peroxidase (POD) in the callus tissues, while also upregulating the expression of key metabolic enzymes such as HMGS, DXR, and ASS-1. These changes suggest that W-1 and W-15 can promote the accumulation of agarwood-related compounds by activating antioxidant defense mechanisms and secondary metabolic pathways. Moreover, GC-MS analysis revealed a significant increase in the content of sesquiterpenes and aromatic compounds in the samples treated with W-1 and W-15, further validating the effectiveness of these two fungi in inducing agarwood formation. Therefore, this study demonstrates that using callus tissue as a method for rapidly screening fungi that promote agarwood formation is feasible and effective. This method not only offers new insights for screening fungi resources that efficiently induce agarwood formation but also contributes to the protection and sustainable use of wild agarwood resources.”

We hope, with these modifications, the quality of our manuscript would meet the publication standard of the Journal. We appreciate very much for your time in editing our manuscript, and the detailed and useful comments and suggestions from you and reviewers. I am looking forward to hearing from your suggestion about our revised manuscript.

Sincerely,

Zhikai Wang

Central South University of Forestry and Technology

College of Life Sciences and Technology

No. 498, Shaoshan South Road, Tianxin District, Changsha City, Hunan Province

[wwzk621@163.com](mailto:wwzk621@163.com)

+86-18339259117
