# Supplementary material for: Research on using Aquilaria sinensis callus to evaluate the agarwood-inducing potential of fungi
Source: PLoS One. 2024 Dec 26;19(12):e0316178. doi: 10.1371/journal.pone.0316178 (PMC11671001; doi:10.1371/journal.pone.0316178)
Supplement: Attachment — Submitted filename: Rebuttal letter.docx [file pone.0316178.s006.docx]

Dear editor,

Thank you very much for your kind letter and the reviewers’ comments concerning our manuscript entitled 'Research on Using Aquilaria sinensis Callus to Evaluate the Agarwood-Inducing Potential of Fungi' (ID: PONE-D-24-13425). The comments were very valuable and helpful for revising and improving our paper as well as for guiding our research. We have thoroughly considered the comments and substantially revised our manuscript. We have highlighted the revisions in the manuscript in red.

**The point-by-point replies to the editor's comments are listed below.**

**Editor's Comments & Our Responses:**

Please review your reference list to ensure that it is complete and correct. If you have cited papers that have been retracted, please include the rationale for doing so in the manuscript text, or remove these references and replace them with relevant current references. Any changes to the reference list should be mentioned in the rebuttal letter that accompanies your revised manuscript. If you need to cite a retracted article, indicate the article’s retracted status in the References list and also include a citation and full reference for the retraction notice.

**Response:** Thank you for the comment. We have checked the references and ensured their completeness and accuracy! Additionally, we have added references to the discussion section according to the reviewer's suggestions and ensured their completeness and accuracy! Here are the additional references we added, located at lines 229, 232, 233, 234, 349, 351, and 252 of the article. Their respective numbers are 31, 32, 33, 34, 36, 38, 39, and 40.

“Fungi serve as natural inductors, facilitate the accumulation of agarwood in a process that is both economically feasible and environmentally sustainable. However, only 8% of the fungi isolated from *Aquilaria* trees have been investigated for their capacity to enhance the production of agarwood [30, 31].

When plants are under stress, it leads to an explosion of ROS that disrupt the plant's internal balance [32]. Plants signal through hormones and ions to activate the downstream defense systems, such as antioxidant enzymes and secondary metabolite synthesis [33]. Finally, a large quantity of stored carbohydrates are synthesized into secondary metabolites [34], thereby assisting the plant in resisting stress.

In the process of agarwood formation, mechanical damage or other stressors activate specific genes and metabolic pathways, which ultimately result in the production of secondary metabolites like sesquiterpenes and 2-(2-phenylethyl) chromone [38]. The metabolic pathway of terpenoids has been extensively researched, but the biosynthesis of chromone derivatives has received relatively less attention [39]. Sesquiterpenes are synthesized by multiple enzymes via the MVA pathway in the cytoplasm and the MEP pathway in the plastids [40].

31.	Zhang X, Wang LX, Hao R, Huang JJ, Zargar M, Chen MX, et al. Sesquiterpenoids in Agarwood: Biosynthesis, Microbial Induction, and Pharmacological Activities. J Agric Food Chem. 2024;72(42):23039-52. Epub 2024/10/08. doi: 10.1021/acs.jafc.4c06383. PubMed PMID: 39378105.

32.	Chen Q, Jin Y, Guo X, Xu M, Wei G, Lu X, et al. Metabolomic responses to the mechanical wounding of Catharanthus roseus' upper leaves. PeerJ. 2023;11:e14539. Epub 2023/03/28. doi: 10.7717/peerj.14539. PubMed PMID: 36968002; PubMed Central PMCID: PMCPMC10035419.

33.	Ghorbel M, Brini F. Hormone mediated cell signaling in plants under changing environmental stress. Plant Gene. 2021;28:100335. doi: 10.1016/j.plgene.2021.100335.

34.	Cui Z, Li X, Xu D, Yang Z. Changes in Non-Structural Carbohydrates, Wood Properties and Essential Oil During Chemically-Induced Heartwood Formation in *Dalbergia odorifera*. Front Plant Sci. 2020;11:1161. Epub 2020/09/10. doi: 10.3389/fpls.2020.01161. PubMed PMID: 32903589; PubMed Central PMCID: PMCPMC7438546.

36.	Liu T, Liu Y, Fu Y, Qiao M, Wei P, Liu Z, et al. Structural, defense enzyme activity and chemical composition changes in the xylem of *Aquilaria sinensis* during fungus induction. Industrial Crops and Products. 2024;208:117804. doi: 10.1016/j.indcrop.2023.117804.

38.	Zhang Z, Zhang X, Yang Y, Wei J-h, Meng H, Gao Z-h, et al. Hydrogen peroxide induces vessel occlusions and stimulates sesquiterpenes accumulation in stems of *Aquilaria sinensis*. Plant growth regulation. 2014;72:81-7. doi: 10.1007/s10725-013-9838-z.

39.	Xu J, Du R, Wang Y, Chen J. Wound-Induced Temporal Reprogramming of Gene Expression during Agarwood Formation in *Aquilaria sinensis*. Plants (Basel). 2023;12(16). Epub 2023/08/26. doi: 10.3390/plants12162901. PubMed PMID: 37631113; PubMed Central PMCID: PMCPMC10459772.

40.	Khodavirdipour A, Safaralizadeh R, Haghi M, Hosseinpourfeizi MA. Comparative de novo transcriptome analysis of flower and root of Oliveria decumbens Vent. to identify putative genes in terpenes biosynthesis pathway. Front Genet. 2022;13:916183. Epub 2022/08/23. doi: 10.3389/fgene.2022.916183. PubMed PMID: 35991569; PubMed Central PMCID: PMCPMC9386285.”

Finally, we would like to thank you once again for your valuable advice!

**The point-by-point replies to the reviewers’ comments are listed below.**

**Reviewers' Comments & Our Responses:**

**Reviewer #1:**

Although these authors responsed to my comments, the writing still need further impoved，such as genes need to be written in italics.

**Response:** Special thanks to the reviewer for the positive evaluation. We apologize for any errors in the details! We have rechecked the details of the article and modified the format of the genes. Thanks again for your helpful suggestions! Here are some of the changes made:

“The experimental results showed that W-1 and W-15 treatments significantly enhanced the activity of Superoxide dismutase (SOD) and Peroxidase (POD) in the callus tissue and upregulated the expression of 3-hydroxy-3-methylglutaryl-CoA synthase (*HMGS*), 1-deoxy-D-xylulose-5-phosphate reductoisomerase (*DXR*), and sesquiterpene synthase (*ASS-1*). The experimental results showed that W-1 and W-15 treatments significantly enhanced the activity of SOD and POD in the callus tissue and upregulated the expression of *HMGS*, *DXR*, and *ASS-1*.

The glyceraldehyde-3-phosphate dehydrogenase (*GADPH*) gene was used as an internal control. The experiment included three independent biological replicates, and the relative expression levels of *HMGS, DXR*, and *ASS-1* were calculated using the 2^-ΔΔCT^ method [22]. The primers used for qRT-PCR are listed in Table 1”

Finally, we would like to thank you once again for your valuable advice!

**Reviewer #4:**

**1.** Abbreviation of each word should be written once in the text of the introduction of the authors instead of the sod text and its full name.

**Response:** Thank you for the comment. We re-reviewed the entire document and made necessary changes based on your feedback. Thank you again for your valuable suggestions！

**2.** “These changes suggest that W-1 and W-15 can promote the accumulation of agarwood-related compounds by activating antioxidant defense mechanisms and secondary metabolic pathways” On what basis do the authors make this claim?

**Response:** Thank you for the comment. I apologize for making an inaccurate statement. We have reviewed the entire article and made changes to the conclusion section in an effort to make our expression more accurate. The changes we made are in lines 281-282 of the article, and the specific changes are as follows:

“These changes suggest that W-1 and W-15 have similar potential to YMY in inducing agarwood formation.”

Once again, we sincerely apologize for our inaccurate description. Finally, we express our gratitude for your valuable suggestions！

**Reviewer #5:**

Dear Editor!
I see that the authors have addressed reviewer comments in detail. I suggest its publicaiton after the followin minor improvements are made to the paper.

**Response:** Thank you for the positive feedback from the reviewers. We will make necessary revisions to our article based on their comments to meet the requirements for publication. Thanks again for your helpful suggestions!

**1.** Clearly specifying the number of biological replicates and technical replicates used in each experiment can reinforce the reliability of the data.

**Response:** Thank you for the comment. The number of replicates for each experiment, which was three, is detailed in the methods section of the manuscript. Thanks again for your helpful suggestions!

**2.** The discussion could be strengthened by integrating more literature to explain the mechanisms by which the observed gene expression changes lead to agarwood formation. This might include discussing how increased superoxide dismutase (SOD) and peroxidase (POD) activities are linked to downstream processes in secondary metabolite production.

**Response:** Thank you for the comment. Your input is highly valued by us. We have made modifications to the discussion section and included more references. The changes are located at lines 231-235 and 347-252 of the article, and here are the specific changes:

“When plants are under stress, it leads to an explosion of ROS that disrupt the plant's internal balance [32]. Plants signal through hormones and ions to activate the downstream defense systems, such as antioxidant enzymes and secondary metabolite synthesis [33]. Finally, a large quantity of stored carbohydrates are synthesized into secondary metabolites [34], thereby assisting the plant in resisting stress.

In the process of agarwood formation, mechanical damage or other stressors activate specific genes and metabolic pathways, which ultimately result in the production of secondary metabolites like sesquiterpenes and 2-(2-phenylethyl) chromone [38]. The metabolic pathway of terpenoids has been extensively researched, but the biosynthesis of chromone derivatives has received relatively less attention [39]. Sesquiterpenes are synthesized by multiple enzymes via the MVA pathway in the cytoplasm and the MEP pathway in the plastids [40].”

Finally, we would like to thank you once again for your valuable advice!

**3.** While the authors mention a few studies, a more thorough comparison with previous research findings would highlight the novelty and importance of their work. For instance, they could elaborate on how their findings align with or differ from past studies on fungal induction of agarwood.

**Response:** Thank you for the comment. Your input is highly valued by us. We emphasized in our discussion the scarcity of fungal resources with the ability to induce agarwood production, and also highlighted that using callus tissue to judge the fungal ability to promote agarwood formation is not only faster and more convenient, but also helps to protect wild *Aquilaria sinensis*. Our modifications are located at lines 226-229 and 275-276 of the article, and here are the specific changes we made:

“Fungi serve as natural inductors, facilitate the accumulation of agarwood in a process that is both economically feasible and environmentally sustainable. However, only 8% of the fungi isolated from *Aquilaria* trees have been investigated for their capacity to enhance the production of agarwood [30, 31].

This approach not only enables the rapid determination of the ability of fungi to promote agarwood formation in the laboratory, but also contributes to the protection of wild *A. sinensis*.”

Finally, we would like to thank you once again for your valuable advice!

**4.** While the authors have included information about the genes studied (e.g., HMGS, DXR, and ASS-1), explaining in greater detail why these specific genes were chosen (beyond their known functions) could add clarity. Additionally, discussing whether other genes related to secondary metabolite pathways were considered and why they were excluded might strengthen the rationale.

**Response:** Thank you for the comment. Your opinion has been very inspiring to us. The production of agarwood is not controlled by a single metabolic pathway or a single gene expression. It is the result of the combined action of multiple factors, which is why agarwood is so valuable. We explain the reasons for choosing HMGS, DXR, and ASS-1 in the abstract and discussion sections, respectively, located at 61-70 lines and 247-252 lines of the article. Here are the specific details:

“Under fungi induction, the secondary metabolic pathways within agarwood trees are significantly activated, particularly the mevalonate (MVA) pathway and the methylerythritol phosphate (MEP) pathway. 3-Hydroxy-3-methylglutaryl-CoA synthase (*HMGS*) and 1-deoxy-D-xylulose-5-phosphate reductoisomerase (*DXR*) are the key rate-limiting enzymes in the MVA and MEP pathways, respectively [13, 14]. The upregulated expression of these enzymes promotes the increased synthesis of sesquiterpene compounds. Sesquiterpene synthase *(ASS-1)* is a typical wound-induced gene responsible for sesquiterpene formation in agarwood. Studies have found that *ASS-1* expression is barely detectable in healthy *A. sinensis* callus or cell cultures, but its expression significantly increases in wounded tissues or jasmonic acid methyl ester-treated callus, leading to a corresponding increase in sesquiterpene compounds [15].

In the process of agarwood formation, mechanical damage or other stressors activate specific genes and metabolic pathways, which ultimately result in the production of secondary metabolites like sesquiterpenes and 2-(2-phenylethyl) chromone [38]. The metabolic pathway of terpenoids has been extensively researched, but the biosynthesis of chromone derivatives has received relatively less attention [39]. Sesquiterpenes are synthesized by multiple enzymes via the MVA pathway in the cytoplasm and the MEP pathway in the plastids [40].”

Finally, we would like to thank you once again for your valuable advice!

We hope, with these modifications, the quality of our manuscript would meet the publication standard of the Journal. We appreciate very much for your time in editing our manuscript, and the detailed and useful comments and suggestions from you and reviewers. I am looking forward to hearing from your suggestion about our revised manuscript.

Sincerely,

Zhikai Wang

Central South University of Forestry and Technology

College of Life Sciences and Technology

No. 498, Shaoshan South Road, Tianxin District, Changsha City, Hunan Province

[wwzk621@163.com](mailto:wwzk621@163.com)

+86-18339259117
